# Activation of the executioner caspases-3 and -7 promotes microglial pyroptosis in models of multiple sclerosis

**DOI:** 10.1186/s12974-020-01902-5

**Published:** 2020-08-29

**Authors:** Brienne A. McKenzie, Jason P. Fernandes, Matthew A. L. Doan, Laura M. Schmitt, William G. Branton, Christopher Power

**Affiliations:** 1 Department of Medical Microbiology & Immunology, University of Alberta, Edmonton, AB Canada; 2 Department of Medicine, University of Alberta, Edmonton, AB Canada; 3 Neuroscience & Mental Health Institute, University of Alberta, Edmonton, AB Canada; 4 Laboratory Medicine & Pathology, University of Alberta, Edmonton, AB Canada

**Keywords:** Neuroinflammation, Pyroptosis, Caspase, Inflammasome, Immunity, Central nervous system, Regulated cell death, Multiple sclerosis

## Abstract

**Background:**

Pyroptosis is a type of proinflammatory regulated cell death (RCD) in which caspase-1 proteolytically cleaves gasdermin D (GSDMD) to yield a cytotoxic pore-forming protein. Recent studies have suggested that additional cell death pathways may interact with GSDMD under certain circumstances to execute pyroptosis. Microglia/macrophages in the central nervous system (CNS) undergo GSDMD-associated pyroptosis in multiple sclerosis (MS) and its animal model experimental autoimmune encephalomyelitis (EAE) but the contribution of other cell death pathways to this phenomenon is unknown. Herein, we tested the hypothesis that multiple RCD pathways underlie microglial pyroptosis in the context of neuroinflammation.

**Methods:**

A siRNA screen of genes with known RCD functions was performed in primary human microglia to evaluate their role in nigericin-induced pyroptosis using supernatant lactate dehydrogenase activity as a read-out of cell lysis. Activation of apoptotic executioner proteins and their contribution to pyroptosis was assessed using semi-quantitative confocal microscopy, high-sensitivity ELISA, immunoblot, cell lysis assays, and activity-based fluorescent probes. Quantification of pyroptosis-related protein expression was performed in CNS lesions from patients with progressive MS and mice with MOG_35-55_-induced EAE, and in matched controls.

**Results:**

Among progressive MS patients, activated caspase-3 was detected in GSDMD immunopositive pyroptotic microglia/macrophages within demyelinating lesions. In the siRNA screen, suppression of caspase-3/7, caspase-1, or GSDMD expression prevented plasma membrane rupture during pyroptosis. Upon exposure to pyroptotic stimuli (ATP or nigericin), human microglia displayed caspase-3/7 activation and cleavage of caspase-3/7-specific substrates (e.g., DFF45, ROCK1, and PARP), with accompanying features of pyroptosis including GSDMD immunopositive pyroptotic bodies, IL-1β release, and membrane rupture. Pyroptosis-associated nuclear condensation and pyroptotic body formation were suppressed by caspase-3/7 inhibition. Pharmacological and siRNA-mediated inhibition of caspase-1 diminished caspase-3/7 activation during pyroptosis. In mice with EAE-associated neurological deficits, activated caspase-3 colocalized with GSDMD immunopositivity in lesion-associated macrophages/microglia.

**Conclusions:**

Activation of executioner caspases-3/7, widely considered key mediators of apoptosis, contributed to GSDMD-associated microglial pyroptosis under neuroinflammatory conditions. Collectively, these observations highlight the convergence of different cell death pathways during neuroinflammation and offer new therapeutic opportunities in neuroinflammatory disease.

## Background

Regulated cell death (RCD) in the central nervous system (CNS) is a major driver of pathogenesis in neurodegenerative and neuroinflammatory diseases [1]. Among the twelve types of RCD currently recognized [2], apoptosis was the first to be identified and has been the most extensively studied in the CNS. During apoptosis, executioner caspases-3 and -7 (-3/7) are cleaved and activated, leading to extensive intracellular proteolysis, disruption of cellular functions, and non-inflammatory cell death [3–5]. Caspase-3/7 activation is widely considered the defining molecular marker for apoptotic cell death [2].

A growing appreciation has also developed for non-apoptotic inflammatory RCD as a determinant of neurological disease. In particular, the lytic cell death program termed *pyroptosis* (“fiery death”) has emerged as a pivotal cell death mechanism in CNS disease [6]. Like apoptosis, pyroptosis relies upon caspase activation as an initiating event in the cell death program; while apoptosis is initiated by caspases-8 and -9 and executed by caspases-3 and -7, pyroptosis is initiated by the caspase-1 family members (caspases-1 and -11 in mice, caspases-1 and -4 in humans) and executed by the pore-forming protein, gasdermin D (GSDMD), or in certain circumstances, gasdermin E (GSDME) [7]. GSDMD can be upregulated at the transcript and/or protein level in response to pyroptotic stimuli [8, 9] before being cleaved by caspase-1-family proteases within the inflammasome, a cytosolic protein complex that also mediates IL-1β and IL-18 maturation [10–12]. Activated GSDMD translocates to the plasma membrane and assembles multimeric pores that are permeable to small molecules, including ions and inflammasome-associated cytokines (IL-1β and IL-18), but not large molecules such as lactate dehydrogenase (LDH) [10–13]. Due to local changes in osmotic pressure, *pyroptotic bodies* form along the membrane, which swell and eventually rupture catastrophically to cause cell lysis [10–14]. This process releases intracellular alarmins (e.g., heat shock proteins), soluble cytoplasmic proteins (e.g., LDH), and inflammatory mediators (e.g., IL-1β and IL-18) into the extracellular milieu, propagating local inflammation.

Live-cell imaging, scanning electron microscopy, and confocal microscopy have been widely utilized to delineate the temporal progression of GSDMD-mediated pyroptosis [13, 15–17]. While GSDMD is diffusely expressed in macrophages exposed to a priming stimulus alone (e.g., lipopolysaccharide), the addition of a lethal pyroptotic stimulus (e.g., the NLRP3-activating toxin nigericin) causes a distinctive enrichment of GSDMD at the plasma membrane early in pyroptosis [13]. This is followed by the formation of bleb-like pyroptotic bodies, which can be observed by confocal or scanning electron microscopy [13]. In the later stages of pyroptosis, the cell membrane ruptures, leaving a relatively intact nucleus and diffuse GSDMD-immunopositive cellular debris [13]. Live cell imaging has recapitulated these findings, demonstrating that diffuse cytoplasmic GSDMD immunoreactivity gives way to localized aggregates at the plasma membrane within 15 min of nigericin exposure, which corresponds to the appearance of bleb-like membrane protrusions (i.e., pyroptotic bodies) at the cell surface [17]. Likewise, *Salmonella typhimurium* and other NLRC4 inflammasome activators trigger pyroptotic body formation, and these can be seen bursting to release cellular contents using time-lapse confocal microscopy [15]. These studies also utilized electron microscopy to demonstrate that a non-viable pyroptotic corpse remains semi-intact after cell death, characterized by a ruptured membrane, a collapsed actin network, and a highly condensed nucleus that eventually disintegrates [15]. Soluble proteins such as LDH are also released upon catastrophic cell membrane rupture, making supernatant LDH activity a useful molecular confirmation of end-stage lytic cell death.

Pyroptosis has been identified in all major CNS cell types and in multiple neurological diseases, including traumatic brain injury [18, 19], sepsis-associated encephalopathy [20, 21], Alzheimer’s disease [22], and multiple sclerosis (MS) [9, 23]. As a prototypic neuroinflammatory disease, MS offers a salient disease model within which to examine the mechanisms governing pyroptosis in the CNS. Evidence for GSDMD-mediated pyroptosis in human macrophages/microglia in vitro and in MS patient white matter was reported previously [9]; likewise, GSDMD immunoreactivity and pyroptosis have been observed in myeloid-lineage cells in the murine CNS during experimental autoimmune encephalomyelitis (EAE) [9, 23]. Importantly, pyroptosis represents an emerging pharmacological target in neurological disease models such as EAE, with well-validated blood-brain barrier (BBB)-permeable inflammasome inhibitors such as the caspase-1 inhibitor VX-765 and the NLRP3 inflammasome inhibitor MCC950 showing promise in preclinical models of CNS disease [9, 24, 25]. Inhibitors of GSDMD such as necrosulfonamide and disulfiram are also in development [23, 26].

In CNS disease models, apoptosis and pyroptosis are often distinguished immunohistologically based upon the specific caspase that is activated within dying cells, with caspase-3/7 considered indicative of apoptosis, and caspase-1/4/11 indicative of pyroptosis. Molecular interactions between inflammatory and apoptotic caspases have occasionally been demonstrated, with caspase-1 serving as an apical caspase that can directly cleave and activate caspase-3/7 under specific circumstances [27–30]. Nonetheless, a functional role for caspase-3/7 in GSDMD-dependent pyroptosis has not been shown. Likewise, the interplay between different caspases during pyroptosis in the context of neuroinflammation has not been examined, and the molecular mechanisms governing pyroptosis in the CNS remain unclear.

In the current manuscript, the molecular mediators of pyroptosis in human CNS macrophages/microglia were interrogated in vitro and subsequently validated in vivo using EAE as a prototypic model of neuroinflammation. Initial studies utilizing MS white matter revealed co-expression of GSDMD with active caspase-3 in myeloid cells, providing the first evidence of crosstalk between apoptotic and pyroptotic pathways in the CNS. An in vitro screen of caspase and gasdermin family members subsequently revealed that inhibition of caspase-3 and/or -7 prevented pyroptosis in human microglia, mirroring the effect of inhibiting the well-characterized pyroptosis mediators, caspase-1 and GSDMD. Prototypic caspase-3/7 substrates PARP, DFF45, and ROCK1 were also cleaved during pyroptosis, recapitulating signature proteolytic events observed during apoptosis, and highlighting the functional proteolytic activity of caspase-3/7 during pyroptosis. Importantly, siRNA-mediated inhibition of caspase-3 and -7 prevented membrane rupture, nuclear disintegration, cleavage of caspase-3/7 substrates, and pyroptotic body formation. These results were validated in vivo by demonstrating expression of active caspase-3 in GSDMD immunopositive CNS macrophages/microglia in EAE. Collectively, these observations provide the first evidence that active caspase-3/7 cooperate with GSDMD to mediate pyroptosis in the context of neuroinflammation.

## Methods and materials

### Ethics statement

Human fetal tissues were obtained from 17–22-week aborted male and female fetuses that were collected with the written informed consent of the donor, approved by the University of Alberta Human Research Ethics Board (Biomedical) (Pro00027660). The use of autopsied brain tissues (Supplemental Table 1) was approved (Pro0002291) by the University of Alberta Human Research Ethics Board (Biomedical) and written informed consent was received for all samples. Cerebral frontal white matter including demyelinating lesions and normal appearing white matter from MS patients and other disease controls (non-MS) were examined. All animals were housed and monitored on a regular schedule, and experiments were performed according to the Canadian Council on Animal Care and University of Alberta Health Sciences Animal Care and Use Committee guidelines.

### Cell cultures

Primary fetal human microglia were isolated based on differential culture conditions, as previously described [31, 32]. Fetal brain tissues from 17–20-week fetuses were dissected, meninges were removed, and a single cell suspension was prepared through enzymatic digestion for 60 min with 2.5% trypsin and 0.2 mg/mL DNAse I, followed by trituration through a 70-μm cell strainer. Cells were washed twice with fresh medium and plated in T-75 flasks. Cultures were maintained in MEM supplemented with 10% FBS, 2 mM l-glutamine, 1 mM sodium pyruvate, 1× MEM nonessential amino acids, 0.1% dextrose, 100 U/mL Penicillin, 100 μg/mL streptomycin, 0.5 μg/mL amphotericin B, and 20 μg/mL gentamicin. For microglial cells, mixed cultures were maintained for 1–2 weeks, at which point astrocytes and neurons formed an adherent cell layer with microglia loosely attached or free floating in the medium. Cultures were gently rocked for 20 min to resuspend the weakly adhering microglia in medium, which were then decanted, washed, and plated. Purity of cultures was verified by immunofluorescence as previously reported [33].

### Induction of pyroptosis and apoptosis

Microglia were plated in 6-well (Western blot, caspase-3 cell lysate ELISA; 5 × 10^5^ cells/mL), 8-well (immunofluorescence; 5 × 10^4^ cells/mL), or 96-well (DAPI, LDH assay; 2.5 × 10^5^cells/mL) plates for 24 h prior to treatment. Cells were exposed to nigericin (5.0 μM; InvivoGen cat# tlrl-nig), nigericin plus VX-765 (50.0 μM, 4 h pre-treatment; InvivoGen cat# inh-vx765-1), ATP-γ-S [100.0 μM, adenosine 5′-O-(3-thiotriphosphate), a thiophosphorylated phosphatase-resistant form of ATP; catalog no. 11162306001; Sigma-Aldrich], ATP plus VX-765 (as above), staurosporine (5.0μM; Abcam #ab120056) staurosporine plus VX-765 (as above), or vehicle control for 4 h unless otherwise indicated. Supernatants were harvested and stored at − 80 °C.

### siRNA knockdown

Microglia were plated as described above for 24 h prior to transfection. Cells were transfected with 30.0 nM of non-targeting universal negative control siRNA (Integrated DNA Technologies TriFECTa RNAi Kit) or a cocktail of three pre-designed commercially available Dicer-Substrate siRNAs targeting the transcript of interest (10.0 nM for each siRNA) in combination with PrecisionFectin^TM^ (Precision Bio Laboratories, Edmonton, Canada SKU #TF071-500) according to manufacturer’s instructions. Cells were permitted to recover for 24 h, and then exposed to nigericin, ATP, or vehicle control for 24 h unless otherwise indicated.

### List of siRNAs

**Table:** 

siRNA	Sequence
Caspase-3 siRNA 1	5′ rGrArC rGrCrU rArCrU rUrUrU rCrArU rGrCrArGrUrU rUrCrU rUrUrG rCrArU rGrArA rArArG
Caspase-3 siRNA 2	5′ rGrGrA rArUrU rGrArU rGrCrG rUrGrA rUrGrUrUrU rArGrA rArArC rArUrC rArCrG rCrArU
Caspase-3 siRNA 3	5′ rUrCrU rGrUrU rGrArA rGrUrUrUrArC RArArU rUrCrC rUrUrU rGrArU rUrGrU rArArA rCrUrU
Caspase-7 siRNA 1	5′ rGrArA rArUrU rGrArC rUrUrArCrArU rArGrA rUrUrU rArUrC rUrArUrGrUrA rArGrU
Caspase-7 siRNA 2	5′ rGrGrG rCrArA rArUrG rCrArU rArArU rGrUrUrGrUrUrUrArUrUrArUrGrArU rGrCrA
Caspase-7 siRNA 3	5′ rGrUrU rUrUrG rArCrG rUrGrA rUrUrG rUrCrUrUrCrArUrUrA rUrArG rArCrArArUrC rArCrG
GSDME siRNA 1	5′ rGrArU rGrGrA rGrUrA rUrCrUrGrArU rCrUrU
GSDME siRNA 2	5′ rGrCrG rGrUrC rCrUrA rUrUrU rGrArU rGrArA
GSDMD siRNA 1	5′ rCrArArCrCrUrGrUrCrUrArUrCrArArGrGrArCrArGrGrArUr GrUrCrCrUrUrGrArUrArGrA
GSDMD siRNA 2	5′ rArCrUrCrUrGrArCrUrUrGrGrArCrGrUrCrCrArArGrArGrArArGrGr ArCrGrUrCrCrArA
GSDMD siRNA 3	5′ rCrArArUrArArArGrGrUrGrGrCrArUrArCrGrUrUrUrCrCrUrCrGr UrArUrGrCrCrArCrC
Caspase-8 siRNA 1	5′ rGrUrCrArUrGrCrUrCrUrArUrCrArGrArUrUrUrCrArGrAAG
Caspase-8 siRNA 2	5′ rArArGrArUrArArUrCrArArCrGrArCrUrArUrGrArArGrAAT
Caspase-8 siRNA 3	5′ rGrCrCrUrGrCrUrGrArArGrArUrArArUrCrArArCrGrArCTA
Caspase-9 siRNA 1	5′ rArUrCrUrArUrGrArArUrUrCrUrArArGrUrGrArArArUrUTT
Caspase-9 siRNA 2	5′ rArArArUrArUrGrUrCrCrUrGrGrGrGrUrArUrArArArArCTT
Caspase-9 siRNA 3	5′ rArArCrArGrArUrGrCrCrUrGrGrUrUrGrCrUrUrUrArArUTT
Caspase-1 siRNA 1	5′ rGrGrArArGrArCrUrCrArUrUrGrArArCrArUrArUrGrCrAAG
Caspase-1 siRNA 2	5′ rArCrCrUrCrUrUrCrCrCrArGrGrArCrArUrUrArArArArUAA
Caspase-1 siRNA 3	5′ rGrCrArArUrCrUrUrUrArArCrArUrGrUrUrGrArArUrArCCA
Caspase-4 siRNA 1	5′ rGrGrArGrCrArCrCrUrUrCrArUrUrArGrUrArCrArGrCrUTG
Caspase-4 siRNA 2	5′ rCrArCrArGrGrGrArUrGrArArGrGrArGrCrUrArCrUrUrGAG
Caspase-4 siRNA 3	5′ rArGrArGrCrUrGrArArGrArGrArUrCrUrArUrCrCrArArUAA

### Immunohistochemistry

Tissue sections were deparaffinized and hydrated using decreasing concentrations of ethanol. Antigen retrieval was performed by boiling the slides in 0.01 M trisodium citrate buffer (pH 6.0). Endogenous peroxidases were inactivated by incubating sections in 0.3% hydrogen peroxide for 20 min. To prevent nonspecific binding, sections were pre-incubated with Odyssey buffer for 1 h at room temperature prior to overnight incubation with primary antibody. Biotinylated secondary antibodies were applied for 2 h, and immunoreactivity detected using the Vectastain Avidin-Biotin Complex (ABC) kit (Vector Laboratories) with a 3,3′-diaminobenzidine tetrachloride (DAB) peroxidase substrate kit (Vector Laboratories). All slides were imaged using an upright microscope (Axioskop2; Zeiss MicroImaging Inc.).

### FAM-FLICA caspase assay

Microglia were plated as above (96-well plate, 24 h) for analysis by microplate reader. Cells were treated with nigericin, ATP, staurosporine, or vehicle control as indicated and caspase-8 (ImmunoChemistry Technologies, #99), caspase-9 (ImmunoChemistry Technologies, #912), and caspase-3/7 (ImmunoChemistry Technologies, #93) assessed according to manufacturer’s instructions.

### Western blot analyses

Immunoblot analysis of tissue and cell lysates was performed as described previously [33, 34]. Following protein extraction using RIPA buffer (Abcam; ab156034), total protein was quantified using a DC Protein Assay Kit (Bio-Rad; cat# 5000112), then treated with Laemmli buffer (Bio-Rad; Cat#161-0747), and incubated at 95 °C for 8 min. Samples were loaded onto 4–20% Precast SDS-PAGE gels (Bio-Rad; Cat# 456-1094) and run for 65–75 min at 100–120 V. Following electrophoresis, gels were transferred onto 0.2 μm nitrocellulose (Bio-Rad; cat# 1620112) membranes using a BioRad Mini Trans-Blot Wet Transfer system for 45–55 min at 0.12 A. To reduce protein loss during successive wash steps, membranes were immersed in 0.4% paraformaldehyde for 30 min, followed by rinses in PBS [35]. Membranes were blocked for 1 h with Odyssey Blocking Buffer (LICOR; cat#927-40000), followed by overnight incubation at room temperature with primary antibody. Membranes were then washed 3 × 5 min with PBS-T (1× PBS-0.05% Tween-20) and incubated with HRP-conjugated secondary antibody (Jackson ImmunoResearch) for 1 h, followed by 3 × 5 min washes. Membranes were developed with ECL reagent (Thermo Scientific; cat#32132) and imaged using an ImageQuant LAS4000 Biomolecular Imager (GE Life Sciences). Band intensity was quantified using ImageStudioLite and normalized to β-actin.

### List of primary antibodies used in Western blots

**Table:** 

Antibody	Dilution
Cleaved PARP (Cell Signalling Technology #9541)	1:250
Total Caspase 3 (R&D Systems AF-605-NA)	1:200
Cleaved ROCK-1 (Novus NB100-56596)	1:200
Cleaved caspase-3 (Cell Signalling Technology #9661)	1:200
Cleaved caspase-7 (Cell Signalling Technology #9491)	1:200
GSDMDC1 (Sigma-Aldrich WH0079792M1 clone 3F12-1B2)	1:500
Caspase-1 (Abcam AB108362)	1:1000
Caspase-8 (Cell Signaling #9746)	1:1000
Caspase-9 (Cell Signaling #9505)	1:1000
Caspase-4 (Cell Signaling #4450)	1:1000
GSDME (Abcam N-terminal ab21519)	1:500
Beta-actin (Santa Cruz Biotechnology, SC-47778)	1:1000

### LDH assay

LDH activity in cell supernatants was assessed using the LDH-Cytotoxicity Assay Kit II (Abcam, ab65393) according to manufacturer’s instructions. Briefly, microglia were plated in 96-well plates and cultured as indicated above for 24 h before transfection or treatment. Supernatants were harvested and stored at − 80 °C prior to use.

### Caspase-3 Cell Lysate ELISA

Microglia were plated in 6-well plates and exposed to pyroptotic or apoptotic stimuli as indicated. Triplicate wells were harvested and pooled and lysates analyzed using the Human Active Caspase-3 Ser29 ELISA Kit (Abcam; ab181418) according to manufacturer’s instructions.

### Cell culture immunofluorescence

Detection of cellular proteins was performed using immunofluorescence as described previously [33]. Cells were cultured on 180-μm-thick polymer coverslip 8-well plates (μ-Slide ibiTreat plates #80826) and treated as appropriate. After 24 h, cells were fixed using 4% paraformaldehyde. Cells were permeabilized using 0.1% Triton in PBS, blocked using Odyssey blocking buffer, and incubated with primary antibody overnight at 4 °C. Primary antibody binding was detected using AlexaFluor 488 goat anti-mouse IgG (Abcam 1:500); AlexaFluor 647 goat anti-rabbit IgG (1:500); or AlexaFluor 568 goat anti-goat IgG (Abcam, 1:500). Cells were stained with DAPI and mounted using Prolong^TM^ Gold antifade reagent (Invitrogen, #P36934). Slides were imaged using a Wave FX spinning disc confocal microscope (Zeiss) with Volocity 6.3 acquisition and analysis software (Perkin Elmer), and basic contrast enhancement performed using unbiased automatic black-point calculation. Composite z-stack images included 15-20 XY planes over a total vertical distance of 4–6 μm using the Improvision Focus Drive. All cells were imaged using a × 40 oil immersion objective lens and quantification was performed on a single XY plane per field of view.

### List of primary antibodies used for immunofluorescence

**Table:** 

Antibody	Dilution
GSDMDC1 (Sigma-Aldrich WH0079792M1 clone 3F12-1B2)	1:200
Caspase-1 p10 (Santa Cruz #514)	1:200
Cleaved PARP (Cell Signalling Technology #9541)	1:200
Total Caspase-3 (R&D Systems AF-605-NA)	1:200
Cleaved ROCK-1 (Novus NB100-56596)	1:200
Cleaved caspase-3 (Cell Signalling Technology #9661)	1:200
Cleaved caspase-7 (Cell Signalling Technology #9491)	1:200
Cleaved DFF45 (Cell Signalling Technology #9731)	1:200
Cleaved GSDMD (provided by FS)	1:100
MHC Class II (Santa Cruz Biotechnology sc-59318)	1:200

### Cell culture immunofluorescence quantification

For quantification purposes, adjacent fields of view were imaged at × 40 and analyzed using Volocity 6.3 acquisition and analysis software. The intensity of each fluorescent channel was quantified by highlighting each individual cell or nucleus to form a region of interest (ROI) and recording the mean fluorescent intensity (MFI) and area (μm^2^) for each ROI. Cross-sectional area of the nucleus and the cell were also recorded. Images were acquired in a single plane without contrast enhancement or other modifications prior to analysis. Background MFI values from a blank portion of the slide were subtracted from total MFI values for every field of view. To assess whether cells were single- or double-immunopositive for proteins of interest, the threshold MFI for “immunopositivity” was determined to be greater than 3-fold higher than the background MFI. Classification of cells into the stages of pyroptosis was performed manually using the criteria indicated: Stage 0: INTACT (adherent cell, elongated processes, baseline GSDMD expression); Stage 1: ROUNDING (rounded cell, loss of processes, increased GSDMD expression throughout the cell); Stage 2: RING-OF-FIRE (translocation of GSDMD to cell membrane, no pyroptotic bodies); Stage 3: PYROPTOTIC BODIES (formation of one or more discreet GSDMD^+^ membrane blebs); Stage 4: LYSIS (membrane rupture, nucleus intact), and Stage 5: GHOST CELLS (nucleus disintegrated, diffuse residual GSDMD^+^ cell debris). MFI was not measured for Stage 5 cells due to the diffuse nature of the cell debris. Slides were imaged unblinded using a Wave FX spinning disc confocal microscope (Zeiss) with Volocity 6.3 acquisition and analysis software (Perkin Elmer).

### Brain tissue immunofluorescence

For human and mouse brain immunofluorescence studies [34], tissue sections on glass slides were de-paraffinized by incubation for 1 h at 60 °C followed by one 10-min and two 5-min incubations in toluene baths through decreasing concentrations of ethanol to distilled water. Antigen retrieval was performed by boiling in 10.0 mM sodium citrate (pH 6.0). Slides were blocked with HHFH buffer [1.0 mM HEPES buffer, 2% (v/v) horse serum, 5.0% (v/v) FBS, 0.1% (w/v) sodium azide in Hank’s balanced salt solution (HBSS)] for 4 h at room temperature. Slides were incubated with primary antibodies at 4 °C overnight. Primary antibody was removed by PBS washes (5 min × 3) and slides were incubated for 3 min in 0.22 mm filtered 1.0% (w/v) Sudan black in 70% ethanol and washed an additional three times in PBS. Slides were incubated in a mixture of 1:400 fluorescent secondary antibodies for 2 h, washed three times in PBS, stained with DAPI for 10 min, and mounted with Prolong Gold (Invitrogen). Slides were imaged unblinded with an inverted Wave FX spinning disc confocal microscope (Zeiss). To categorize cells as immunopositive for proteins of interest, the threshold MFI for “immunopositive” was established as triple the background MFI for each channel and positive cells manually counted in Volocity 6.3 analysis software as previously described [9].

### Experimental autoimmune encephalomyelitis induction and monitoring

C57BL/6 mice (Jackson Labs; female, 10–12 weeks old; 20.3 ± 0.22 g, housed in standard housing conditions with 5 animals/cage in reusable plastic cages in accordance with institutional guidelines) were immunized with MOG_35-55_ peptide (1 mg/mL) emulsified with complete Freud’s adjuvant (CFA) (Hooke laboratories, EK-0115/EK-2110, Lawrence, MA, USA) and injected with pertussis toxin (200 ng/mouse) at day 0 and day 2 according to the manufacturer’s instructions (*n* = 10 EAE mice) [34]. Controls (*n* = 10) received the same CFA and pertussis toxin administrations but not MOG peptide. EAE induction was performed under temporary isoflurane anesthetic with heat pad recovery in accordance with institutional guidelines. Cages (*n* = 5 animals/cage) were assigned to treatment groups using a random number generator (https://www.graphpad.com/ quickcalcs/randomN1.cfm). For the duration of each experiment, animals were fed a Bio-Serv NutraGel diet (BioServ #F4798-KIT) instead of conventional chow to prevent dehydration and aid wellbeing after onset of disease. EAE animals were assessed daily in late afternoon and scored for disease severity up to 24 days following EAE induction using an established 0–15 point scale [9, 34]. A score of 0 indicated no neurobehavioral signs; a score of 1–2 indicated tail paralysis (moderate or severe); a score of 3–8 indicated tail and hind leg paralysis (of varying degrees of severity, unilateral or bilateral); a score of 9–14 indicated tail, hindlimb, and forelimb paralysis (of varying degrees of severity, unilateral or bilateral); and a score of 15 indicated moribund. Three EAE animals met endpoint criteria for disease severity prior to the end of the experiment and were sacrificed. All remaining animals were sacrificed using cervical dislocation at the day 24 endpoint. Spinal cords were collected for tissue immunofluorescence.

### Statistical analyses

The Chi-square test was used to compare the distributions of microglia across the stages of pyroptosis and the distributions of single- and double-immunopositive macrophages/microglia in tissue. Comparisons between means of two groups were performed by unpaired Student’s *t* test or by ANOVA with Dunnett’s post hoc tests in GraphPad Instat 3.0 (GraphPad Software, San Diego, CA, USA). No sample size calculations were performed.

## Results

### Cleaved GSDMD-immunopositive macrophages/microglia are present in MS lesions

Earlier studies demonstrated GSDMD immunoreactivity localized to macrophages/microglia in normal-appearing white matter (NAWM) of progressive MS patients, representing the first evidence of GSDMD-mediated pyroptosis in the CNS [9]. In the current study, an antibody specific for cleaved (active) GSDMD (kindly provided by Dr. Feng Shao, National Institute of Biological Sciences, Beijing) was used in combination with the previously validated antibody detecting total GSDMD to extend the above findings [36]. The specificity of the cleaved GSDMD antibody for the active peptide has been previously validated [36] and was further confirmed using nigericin-treated wild-type and GSDMD knock-out THP-1 cell lines (kindly provided by Dr. Daniel Muruve, University of Calgary) (Supplemental Figure 1A). Using autopsied brain tissues from patients with progressive MS (Supplemental Table 1), demyelinated frontal white matter (WM) lesions were shown to exhibit myelin loss (Luxol Fast Blue ; LFB), abundant CD68-immunopositive macrophages/microglia, and CD3-immunopositive T lymphocytes (Supplemental Figure 2Aiv–ix). This contrasted with frontal white matter from nonMS patients without neuropathology (“nonMS”) in which LFB staining was preserved with minimal CD68 and CD3 immunopositivity (Supplemental Figure 2Ai–iii).

**Fig. 1:**
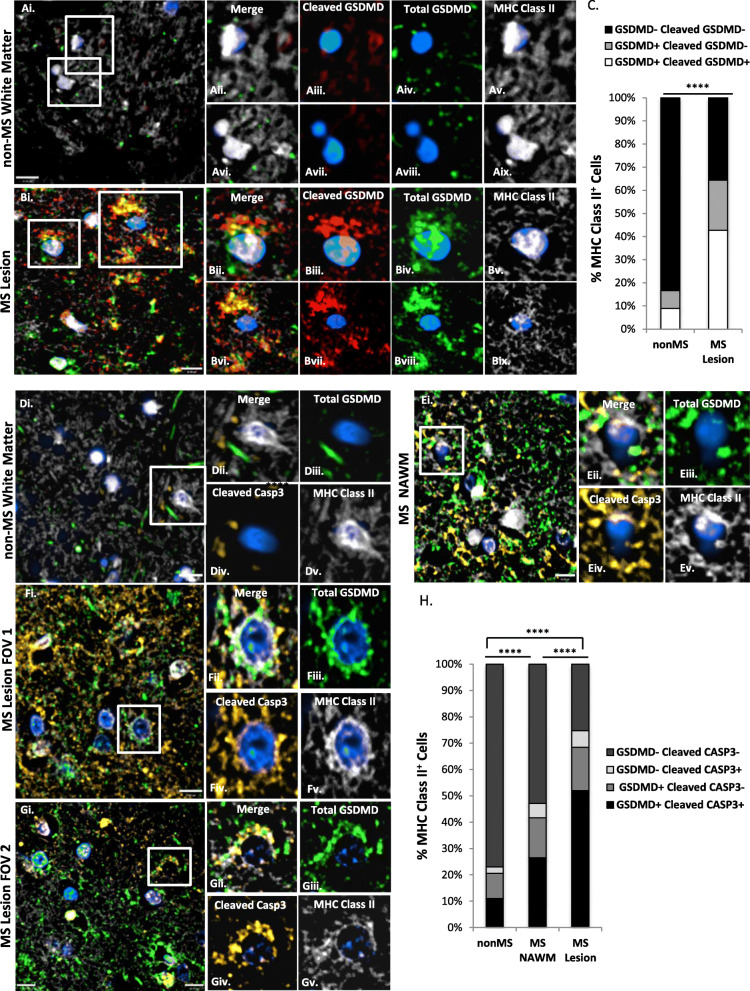
Macrophages/microglia in MS lesions co-express cleaved GSDMD and cleaved caspase-3. (**a**, **b**) Autopsied brain tissue sections from non-MS patients with neuropathologically normal brains (nonMS white matter; **a**) and MS patients with white matter lesions (**b**) were immunolabelled for cleaved GSDMD (red), total GSDMD (green), and MHC class II (white) and imaged using confocal microscopy (representative images are shown). (**c**) Each MHC class II^+^ cell was categorized as single-immunopositive, double-immunopositive, or double-immunonegative for total GSDMD and cleaved GSDMD based on mean fluorescence intensity (MFI), using a threshold of 3X background fluorescence. A total of *n* = 78 MHC class II^+^ cells from 10 unique fields of view (FOV) derived from the neuropathologically normal nonMS control were categorized; *n* = 295 MHC class II^+^ cells from 30 unique FOV derived from two MS patients were categorized. Distribution of cells within each of the three categories was significantly different between nonMS white matter and MS white matter lesions by Chi-square test (****p < 0.0001). Scale bar = 10 μm. (**d**–**g**) Autopsied brain tissue sections for nonMS (**d**) and MS patients (**e**–**g**) were immunolabelled for cleaved caspase-3 (amber), total GSDMD (green), and MHC class II (white) and imaged using confocal microscopy (representative images are shown). (**h**) MHC class II^+^ cells were categorized as single-immunopositive, double-immunopositive, or double-immunonegative for cleaved caspase-3 and GSDMD based on MFI using a threshold of 3X background fluorescence. A total of *n* = 209 MHC class II^+^ cells from 29 unique fields of view (FOV) derived from two neuropathologically normal nonMS controls were categorized; *n* = 223 MHC class II^+^ cells from 25 unique FOV derived from the normal-appearing white matter (NAWM) of two MS patients were also categorized, along with *n* = 554 MHC class II^+^ cells from 58 unique FOV within MS lesions. Distribution of cells within each of the four categories was significantly different between nonMS and both MS NAWM and MS lesions by Chi-square test (****p < 0.0001). Scale bar = 10 μm

To quantify active versus total GSDMD immunoreactivity in MS lesions, confocal microscopy was utilized to detect cleaved/active (red) and total (green) GSDMD, using major histocompatibility complex (MHC) class II as a marker for macrophages/microglia (white) (Fig. 1(A–C)). Of note, the number of MHC class II immunopositive cells per field of view (FOV) was significantly increased in MS lesions compared to nonMS brains (Supplemental Figure 2B). In MS lesions, 42.7% of macrophages/microglia were immunopositive for both total and cleaved GSDMD, while a further 21.7% were positive for total but not cleaved GSDMD; by contrast, over 83.3% of macrophages/microglia in non-MS white matter were double-immunonegative for either total or cleaved GSDMD (Fig. 1(C)). The difference between these two distributions was statistically significant (*p* < 0.0001). The number of macrophages/microglia per FOV that were double immunopositive for total and cleaved GSDMD was also significantly increased in MS lesions compared to nonMS brains (*p* < 0.0001) (Supplemental Figure 2C). By demonstrating that the majority (66.3%) of GSDMD immunopositive microglia/macrophages in MS lesions contained active GSDMD, the present results confirm and extend previous reports of GSDMD in MS lesions [9], and provided further evidence for pyroptosis in the CNS during neuroinflammation.

### Cleaved caspase-3 is co-expressed with GSDMD in microglia/macrophages within MS lesions

We next investigated whether cleaved caspase-3 was detectable in GSDMD immunopositive microglia/macrophages. In frontal white matter from nonMS patients (Fig. 1(D, H)), the majority (77%) of MHC class II immunopositive macrophages/microglia (white) were double immunonegative for cleaved caspase-3 (amber) and GSDMD (green), with only 11% expressing cleaved caspase-3 and GSDMD. Conversely, in active demyelinating MS lesions, 52% of MHC class II immunopositive macrophages/microglia co-expressed both GSDMD and cleaved caspase-3 (Fig. 1(F, H)). A subset of these double-positive macrophages/microglia appeared to display prototypic GSDMD immunopositive pyroptotic bodies (Fig. 1(Fii–iii)). Normal-appearing white matter (NAWM) from matched MS patients expressed an intermediate phenotype, with 27% of macrophages/microglia being double-immunopositive for GSDMD and cleaved caspase-3 (Fig. 1(E, H)). The differences between these three distributions were statistically significant (*p* < 0.0001). The number of macrophages/microglia per FOV that were double immunopositive for GSDMD and cleaved caspase-3 was also significantly increased in MS lesions compared to both NAWM (*p* < 0.001) and nonMS brains (*p* < 0.0001) (Supplemental Figure 2D), and in NAWM compared to nonMS controls (*p* < 0.0001). Further to these observations, double immunopositive cellular remnants with disintegrated nuclei were also apparent within MS lesions, recapitulating the phenotype of end-stage pyroptotic cell death (Fig. 1(Gii–v)). Collectively, these observations demonstrated that activation of apoptotic caspase-3 occurs in pyroptotic macrophages/microglia during neuroinflammation.

### Pyroptotic human microglia display cleaved GSDMD in vitro

**Fig. 2:**
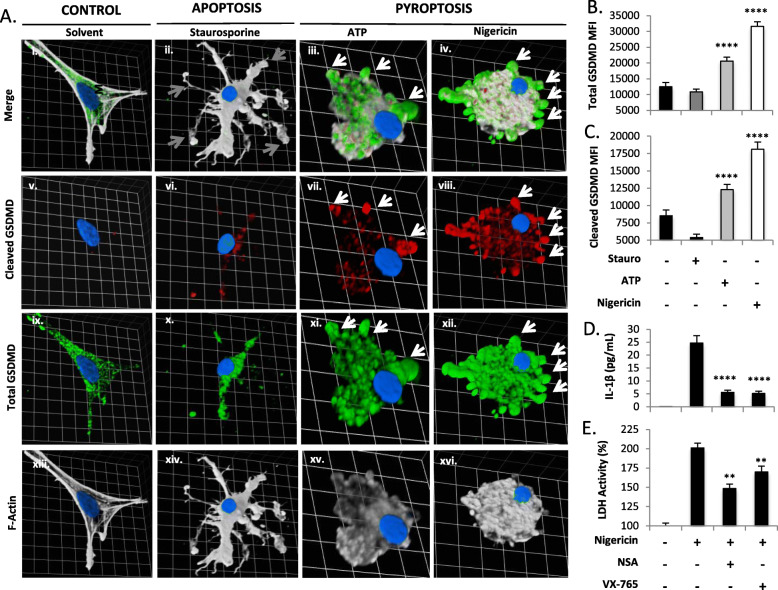
Cleaved GSDMD is present in human microglia during pyroptosis. Primary human microglia were exposed to the pro-pyroptotic stimulus ATP (100.0 μM, 24 h), the pro-pyroptotic stimulus nigericin (5.0 μM, 4 h), the pro-apoptotic stimulus staurosporine (5.0 μM, 4 h), or vehicle [equivalent volume PBS (24 h)]. Cells were fixed, immunolabelled for cleaved GSDMD (Av–viii, red) and total GSDMD (Aix–xii, green), labeled with F-actin (Axiii–Axvi, white; merge shown in Ai–iv), and visualized by confocal microscopy. Images represent three-dimensional z-stacks incorporating 15 XY planes over a vertical distance of 4–6 μm. One square unit represents 10.28 μm. (**b**, **c**) Mean fluorescence intensity (MFI) of each protein was assessed for a minimum of *n* = 20 microglia per condition. Cell supernatants from microglia exposed to nigericin, ATP, or staurosporine as in (**a**) were used to measure IL-1β release (**d**) and LDH activity (**e**) in each condition. Data shown represent mean ± SEM for a representative human donor. Data were tested for significance using one-way ANOVA with Dunnett’s test for multiple comparisons (**p < 0.01, ****p < 0.0001). All experiments were recapitulated in microglia derived from two to three separate human donors

Exposure of cultured human microglia to the established NLRP3 inflammasome activators, nigericin (a bacterial toxin that causes K^+^ efflux) or extracellular ATP, has been previously shown to cause pyroptosis, indicated by IL-1β release, formation of GSDMD immunopositive pyroptotic bodies, and membrane rupture [9]. Pyroptosis was prevented by either pharmacological inhibition of caspase-1 or siRNA-mediated knockdown of GSDMD [9]. In the present studies, the expression of cleaved GSDMD was examined in primary human microglia upon exposure to either nigericin or exogenous ATP by confocal microscopy. Quantification of mean fluorescence intensity (MFI), defined as average fluorescence intensity per unit area, verified that microglia exposed to pyroptosis-inducing stimuli in vitro were immunopositive for both cleaved and total GSDMD, while cells exposed to vehicle alone, or the apoptosis-inducing stimulus, staurosporine, displayed minimal cleaved GSDMD immunoreactivity (Fig. 2(A–C)). Pyroptotic bodies were highly immunopositive for both cleaved and total GSDMD (white arrows, Fig. 2(Avii, viii, xi, xii)), consistent with the role of GSDMD pores in the formation of these structures. As expected in this system, pre-treatment of nigericin-exposed microglia with VX-765 (a well-characterized caspase-1 inhibitor [9, 37, 38]) or necrosulfonamide (which blocks both GSDMD oligomerization [26] and caspase-1 activation [39]) reduced IL-1β release and inhibited cell lysis as measured by supernatant LDH activity (Fig. 2(D, E)).

### Caspase-3 and -7 mediate pyroptosis in human microglia

**Fig. 3:**
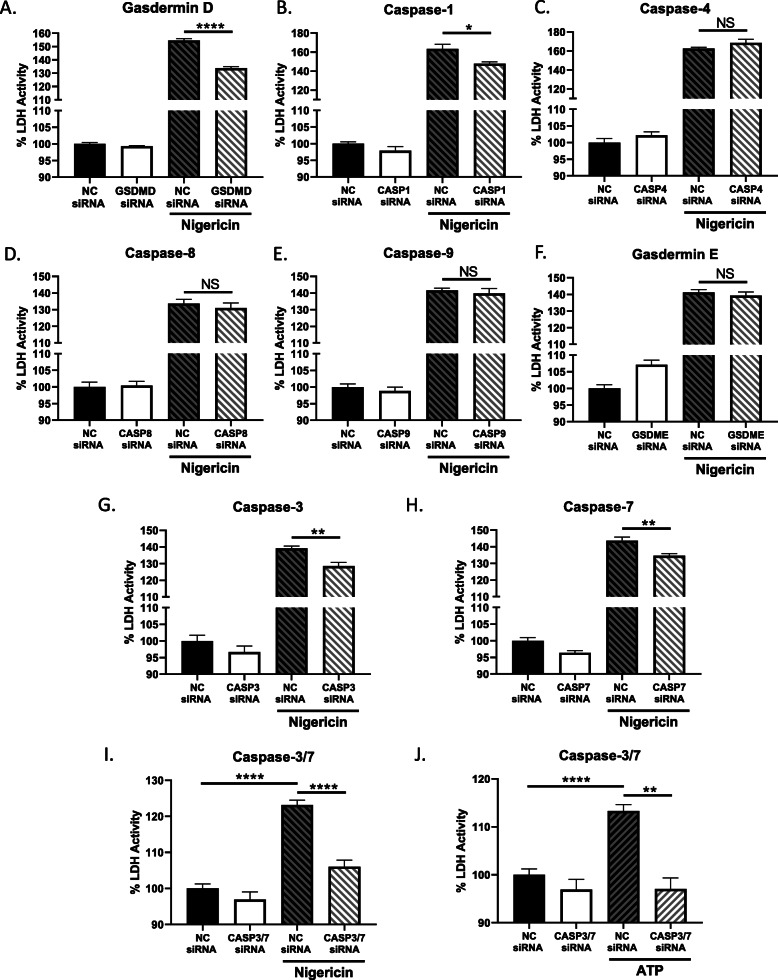
A siRNA screen in human microglia revealed that caspase-3/7 knockdown inhibits LDH release associated with pyroptosis. **a**–**h** Microglia were transfected with either universal non-coding siRNA (NC siRNA) or a cocktail of three different siRNAs targeting GSDMD (**a**), caspase-1 (**b**), caspase-4 (**c**), caspase-8 (**d**), caspase-9 (**e**), GSDME (**f**), caspase-3 (**g**), or caspase-7 (**h**) prior to nigericin exposure (5 μM, 4 h) and cell supernatants were measured for LDH activity. To account for overlapping substrate profiles of caspase-3 and -7, supernatant LDH activity was measured in microglia transfected with either universal non-coding siRNA (NC siRNA) or a cocktail of six different siRNAs targeting caspase-3 and -7 prior to nigericin (5 μM, 4 h) (**i**) or ATP (100.0 μM, 24 h) (**j**) exposure. Data shown represent mean MFI ± SEM for a representative human donor (*p < 0.05, **p < 0.01, ****p < 0.0001)

Next, a siRNA screen of caspase and gasdermin family members with known RCD functions was performed, in order to evaluate their role in the context of nigericin-induced pyroptosis using supernatant LDH activity as a read-out of cell lysis. All siRNAs utilized in this screen were validated by immunoblot (Supplemental Figure 3A–O). Consistent with previous observations [9], siRNA-mediated knockdown of GSDMD inhibited pyroptosis-associated membrane lysis, as indicated by reduced supernatant LDH activity (Fig. 3a). Consistent with its well-known role as an initiator of pyroptosis, knockdown of caspase-1 also reduced LDH release (Fig. 3b). Caspase-4 and GSDME both have roles in pyroptosis under certain circumstances [40], but neither was shown to be involved in LDH release in this model system (Fig. 3c, f). Caspases-8 and -9, which are well-recognized apoptosis initiator caspases, likewise did not contribute to LDH release in nigericin-exposed microglia (Fig. 3d, e). In contrast, siRNA-mediated inhibition of caspase-3 or -7 significantly reduced LDH release (Fig. 3g, h). Since caspases-3 and -7 have overlapping functions and substrate profiles, we targeted both caspases simultaneously to test whether dual inhibition would more fully inhibit cell lysis. Indeed, simultaneous inhibition of caspase-3/-7 effectively abolished nigericin-induced LDH release (Fig. 3i). To ensure this effect was not nigericin-specific, these results were recapitulated during ATP-induced pyroptosis in microglia (Fig. 3j).

### Cleavage of caspase-3/7 and their substrates occurs in microglial pyroptosis

**Fig. 4:**
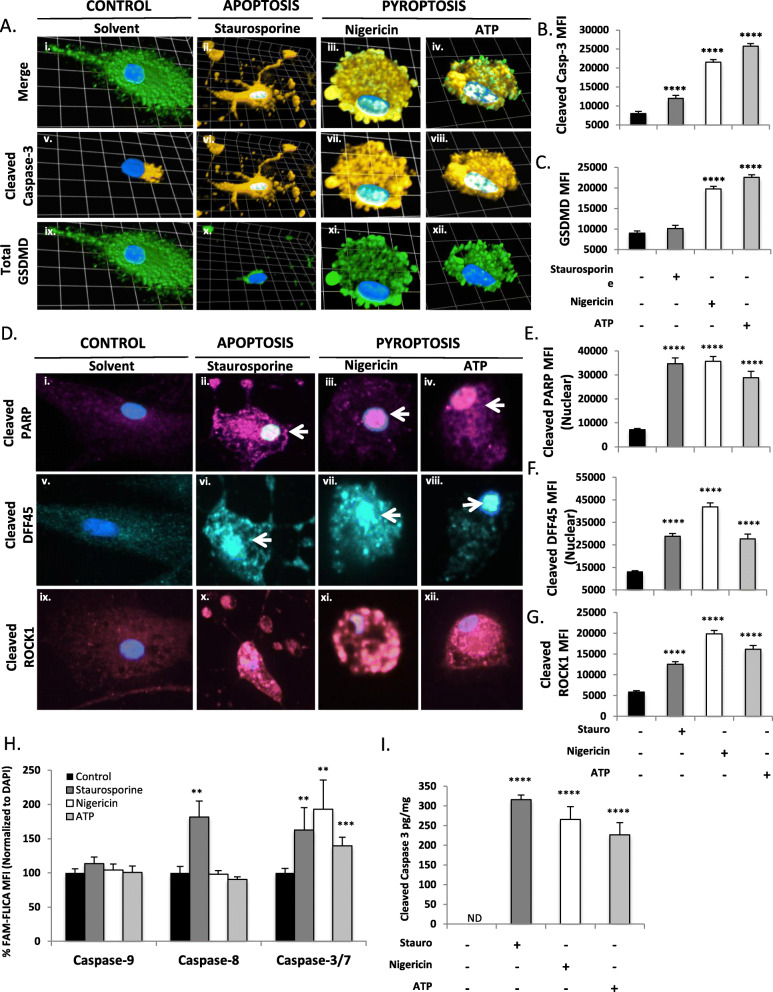
Caspase-3 and its substrates are cleaved during pyroptosis in microglia. Primary human microglia were exposed to the pro-pyroptotic stimuli ATP (100.0 μM, 24 h) or nigericin (5.0 μM, 4 h), the pro-apoptotic stimulus staurosporine (5.0 μM, 4 h), or vehicle [equivalent volume PBS (24 h)]. Cells were fixed and immunolabelled for cleaved caspase-3 p17/p19 (Av–viii, amber) and total GSDMD (Aix–-xii, green), merge shown in (Ai–iv), and visualized by confocal microscopy. Images represent three-dimensional z-stacks incorporating 15 XY planes over a vertical distance of 4–6 μm. One square unit represents 10.28 μm. Human microglia immunolabelled for cleaved caspase-3 p17/p19 (**b**) and GSDMD (**c**) were visualized using confocal microscopy. Mean fluorescence intensity (MFI) of each protein was assessed for a minimum of *n* = 40 microglia per condition. Data shown represent mean MFI ± SEM for a representative human donor. These data were recapitulated in microglia from three separate human donors. Microglia were similarly exposed to staurosporine (5 μM, 4 h), nigericin (5 μM, 4 h), or ATP (100 μM, 24 h), fixed, and immunolabelled for cleaved PARP (Di–iv, magenta), cleaved DFF45 (Dv–viii, cyan), or cleaved ROCK1 (Dix–xii, pink). Representative two-dimensional images shown (nuclei indicated by white arrows). Nuclear mean fluorescence intensity (MFI) of cleaved PARP (**c**) and cleaved DFF45 (**d**) was assessed (*n* = 10–20 cells per condition). Total MFI for cleaved ROCK1 was also assessed (**g**). Microglia in stage 0 were excluded from analysis. Data shown represent mean MFI ±- SEM for a representative human donor. Data were tested for significance using one-way ANOVA with Dunnett’s test for multiple comparisons (****p < 0.0001). To identify caspase activation following pyroptotic or apoptotic stimuli, microglia were exposed to ATP (100.0 μM, 24 h), nigericin (5.0 μM, 4 h), and staurosporine (1 μM, 4 h) and caspase-9, -8 and -3/7 activity was assessed using activity-dependent caspase fluorescent probes and normalized to DAPI (**h**). Microglia were exposed to pyroptotic stimuli ATP (100.0 μM, 24 h) or nigericin (5.0 μM, 4 h), from which lysates were harvested and cleaved caspase-3 levels assessed by ELISA (**i**). Staurosporine exposure (1 μM, 4 h) was utilized as a positive control for caspase-3-activation. Data shown represent mean cleaved caspase-3 levels ± SEM (*n* = 6 technical replicates) from a representative human donor. Results were confirmed in microglia from 3 to 5 donors

To define both the expression and sub-cellular localization of cleaved caspase-3/7 at the single-cell level, we assessed cleaved caspase-3 and GSDMD immunoreactivity in pyroptotic microglia using the apoptotic stimulus staurosporine as a positive control (Fig. 4(A); cleaved caspase-3 amber; GSDMD green). As shown previously (Fig. 2(A)), ATP- and nigericin-exposed microglia recapitulate the signature pyroptotic morphology characterized by highly GSDMD immunopositive pyroptotic bodies, while staurosporine-exposed apoptotic microglia displayed long protruding apoptopodia with minimal GSDMD immunoreactivity (Fig. 4(A)). Cleaved caspase-3 immunoreactivity (amber) was evident in staurosporine-, nigericin-, and ATP-exposed microglia (Fig. 4(Av–viii, B)), particularly in the nucleus and pyroptotic bodies. Quantification of MFI confirmed that both pro-apoptotic and pro-pyroptotic stimuli significantly enhanced cleaved caspase-3 immunoreactivity (Fig. 4(B)), but GSDMD MFI was selectively increased under pyroptotic conditions (*p* < 0.0001) (Fig. 4(Aix–xii, C)). Cleaved caspase-7 immunoreactivity also increased significantly in both nigericin- and ATP-exposed microglia (Supplemental Figure 4).

To determine whether caspase-3/7 were proteolytically active during microglial pyroptosis, we assessed whether prototypic substrates of caspase-3/7 (e.g., PARP, DFF45, and ROCK1) were cleaved following nigericin or ATP exposure. Cleavage of these substrates was assessed by confocal microscopy using antibodies that selectively recognized the cleaved peptides (Fig. 4(Di–xii)). Quantification of MFI indicated a significant intracellular accumulation of the cleaved peptides during both pyroptosis and apoptosis (Fig. 4(E–G)) (*p* < 0.0001). These findings highlighted the proteolytic activity of caspase-3/7 during microglial pyroptosis. To determine the effects of caspase-3/7 inhibition on the accumulation of cleaved caspase-3/7 substrates, cleavage of PARP (Supplemental Figure 5A, B), DFF45 (Supplemental Figure 5C, D), and ROCK1 (Supplemental Figure 5E, F) was assessed following nigericin exposure in the presence of caspase-3/7-targeting siRNAs. Caspase-3/7 inhibition rescued PARP, DFF45, and ROCK1 cleavage, confirming that processing of these substrates was dependent on caspase-3/7 activation as expected.

To verify the activity of caspase-3/7 following exposure to pro-pyroptotic stimuli, we utilized activity-dependent caspase fluorescent probes (FAM-FLICA™ caspase assays) to screen for enzymatically active caspase-3/7, caspase-8, or caspase-9 following exposure to apoptotic and pyroptotic stimuli. While neither caspase-8 nor -9 activity was detected in nigericin- or ATP-exposed microglia, caspase-3/7 activity was significantly (*p* < 0.01) induced under both apoptotic and pyroptotic conditions (Fig. 4(H)).

To confirm the above findings, the presence of activated caspase-3 in staurosporine-, nigericin-, and ATP-exposed microglial lysates was investigated by highly sensitive ELISA (Fig. 4(I)). A significant (*p* < 0.0001) increase in intracellular cleaved caspase-3 concentration was apparent under both apoptotic and pyroptotic conditions.

As a negative control, microglia were exposed to exogenous inflammasome-associated cytokines (IL-1β and IL-18), which activate microglia but do not induce pyroptosis (Supplemental Figure 6A–D). Following cytokine exposure, microglia remained intact and adherent (Supplemental Figure 6A), and LDH was not released (Supplemental Figure 6C, D). Consistent with the hypothesis that caspase-3 is only activated in response to lethal stimuli, active caspase-3 was not detected in IL-1β- or IL-18-exposed microglia despite being abundant in nigericin-exposed cells (Supplemental Figure 6A, B). Taken together, these results indicated that microglia activate caspase-3 and -7 during pyroptosis, but not under non-lethal proinflammatory conditions.

### Caspase-3/7 cleavage is observed in multiple stages of pyroptosis

**Fig. 5:**
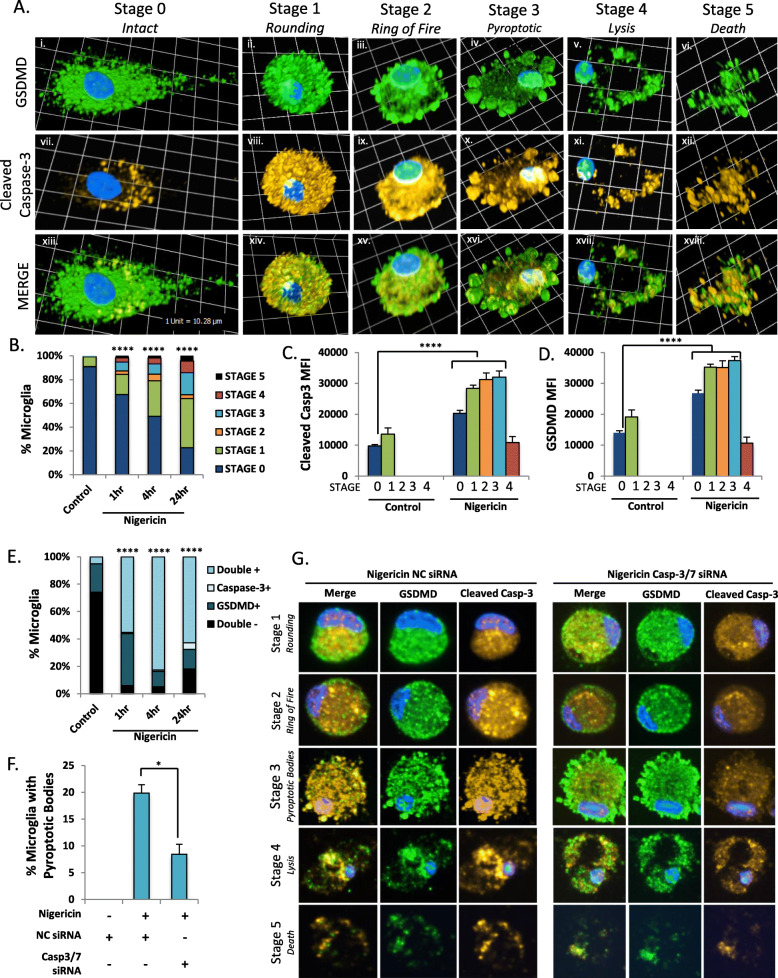
Activated caspase-3 mediates pyroptotic body formation and cell death. Microglia were exposed to nigericin (5.0 μM, 4 h), fixed, and immunolabelled for GSDMD (Ai–vi, green) and cleaved caspase-3 p17/p19 (Avii–xiii, amber), merge shown in (Axiii–xviii) and visualized by confocal microscopy. Based upon morphological and molecular characteristics, five stages of pyroptosis were defined including Stage 0: “Intact” (adherent cell with elongated processes, baseline GSDMD expression); Stage 1: “Rounding” (rounded cell, loss of processes, increased GSDMD expression throughout the cell); Stage 2: “Ring-of-fire” (translocation of GSDMD to the cell membrane); Stage 3: “Pyroptotic bodies” (formation of GSDMD^+^ membrane blebs); Stage 4: “Lysis” (rupture of the cell, nucleus still intact), and finally Stage 5: “Ghost cells” (nucleus disintegrated, residual GSDMD^+^ cell debris). Images shown for each stage are representative three-dimensional z-stacks incorporating 15 XY planes over a vertical distance of 4–6 μm. (B) The proportion of cells at each stage was quantified at 0, 1, 4, and 24 h post-exposure to nigericin, with a minimum of *n* = 60 unexposed microglia and *n* = 100 microglia at each time point post-exposure. Time course data represent the proportion of cells at each stage for a representative sample and have been replicated in microglia derived from two independent human donors. Distribution of the cells across the stages was assessed using a Chi-squared test. (C, D) Microglia from (A) were classified according to the stage of pyroptosis and cleaved caspase-3 (C) or total GSDMD (D) MFI was assessed for each stage of pyroptosis at 4 h post exposure to nigericin. All time course data were independently replicated using microglia derived in 2–3 human donors. To determine co-localization of caspase-3 and GSDMD, human microglia were exposed to nigericin (5 μM, 1, 4, or 24 h), fixed, immunolabelled for cleaved caspase-3 and GSDMD (E). Mean fluorescence intensity (MFI) of each protein was assessed. Each cell was categorized as single-immunopositive, double-immunopositive, or double-immunonegative for GSDMD and cleaved caspase-3 using a threshold of 3X background fluorescence. A minimum of *n* = 40 microglia were classified per time point. Distribution of cells within each of the four categories was significantly different from control using Chi-square test (****p < 0.0001). (F–G) Microglia were transfected with either universal non-coding siRNA (NC siRNA) or a cocktail of different siRNAs targeting caspases-3 and -7 (Casp-3/7 siRNA) prior to nigericin exposure (5 μM, 24 h). Cells were fixed, immunolabelled for GSDMD (green) and cleaved caspase-3 (amber), and the proportion of cells at each stage was quantified. A minimum of *n* = 100 cells were quantified in each condition and results recapitulated in two independent human donors

Several morphologically distinct stages of pyroptosis have previously been observed using live-cell, confocal, and electron microscopy; these include cellular rounding/activation, enrichment of GSDMD at the cell surface, formation of pyroptotic bodies, and rupture of the plasma membrane culminating in cell lysis and the formation of the residual pyroptotic corpse [13, 15, 17]. For the purposes of this study, we have delineated these stages as follows: Stage 0: “Intact” (adherent cell, elongated processes, baseline GSDMD expression); Stage 1: “Rounding” (rounded cell, loss of processes, increased GSDMD expression throughout the cell); Stage 2: “Ring-of-fire” (enrichment of GSDMD at the plasma membrane); Stage 3: “Pyroptotic bodies” (formation of GSDMD^+^ membrane blebs); Stage 4: “Lysis” (membrane rupture, nucleus condensed but intact), and Stage 5: “Ghost cells” (nucleus disintegrated, residual GSDMD^+^ cell debris). Illustrative examples of each of these stages are shown in Fig. 5(Ai–vi).

Before examining caspase-3/7 in this setting, microglia were profiled at different time points following nigericin exposure, revealing that the proportion of intact (Stage 0) microglia decreased with time, while the proportion of cells undergoing pyroptosis at different stages increased (Fig. 5(B)). By contrast, microglia exposed to non-lethal concentrations of exogenous IL-1β and IL-18 remained in Stage 0 (Supplemental Figure 6E). As caspase-1 activation is upstream of GSDMD activation, we hypothesized that caspase-1 inhibition with VX-765 would prevent activation and translocation of GSDMD to the plasma membrane, which would be apparent as a decrease in the number of cells in Stage 2 (“Ring-of-fire”) and thereafter (Supplemental Figure 7A). Indeed, caspase-1 inhibition by VX-765 significantly (*p* < 0.01) altered the distribution of cells within the different stages of pyroptosis, and inhibited progression into Stage 2, reducing the accumulation of GSDMD at the plasma membrane (Supplemental Figure 7B). This was associated with a reduction in cleaved but not total GSDMD MFI (Supplemental Figure 7C–E). These observations provided validation for the present classification of the stages of pyroptosis.

Predicated on this molecular and morphological classification, the time- and stage-dependent accumulation of cleaved caspase-3/7 was assessed following nigericin exposure. Cleaved caspase-3 (amber) was expressed at very low levels in resting Stage 0 microglia, but abundantly expressed during activation (Stage 1) and pyroptosis (Stage 2 onwards), with particularly abundant expression in the nucleus and in pyroptotic bodies (Fig. 5(Avii–xii)). Quantification of cleaved caspase-3/7 by stage revealed that MFI peaked in Stages 1–3 (“Rounding,” “Ring-of-fire,” and “Pyroptotic bodies”) before decreasing during Stage 4 (“Lysis”) (Fig. 5(A, C) and Supplemental Figure 8A–D). The confirmation of cleaved caspase-3/7 at Stages 2 and 3 was particularly pertinent because these stages display unequivocal morphological indicators of pyroptosis that are not shared with other RCD programs. Consistent with previous observations [9], GSDMD MFI also increased significantly (*p* < 0.0001) during pyroptosis, with expression peaking at Stages 1–3 and declining thereafter (Fig. 5(A, D) and Supplemental Figure 8E, F).

To verify our observation that GSDMD and cleaved caspase-3 were indeed co-expressed in the same cells during microglial pyroptosis (Fig. 5(A)), each cell quantified above was classified as single-immunopositive, double-immunopositive, or double-immunonegative based on cleaved caspase-3 and GSDMD expression. Double-immunopositive cells were rare in untreated cells but predominated at all time points tested following nigericin exposure (Fig. 5(E)). Importantly, the cleaved caspase-3 single-immunopositive cell population was minimal at all time points following nigericin exposure (Fig. 5(E)). Collectively, these data supported the presence of cleaved caspase-3 in GSDMD immunopositive cells undergoing pyroptosis, rather than in a subset of cleaved caspase-3 single-positive apoptotic microglia.

### Activation of caspase-3/7 mediates pyroptotic body formation during pyroptosis

The localization of caspase-3 at the plasma membrane and within pyroptotic bodies following nigericin exposure (Fig. 5(A)) suggested a putative role for caspase-3/7 or their substrates in pyroptotic body formation. To determine whether caspase-3/7 played a functional role in the formation of these membrane structures, the proportion of cells in each stage of pyroptosis was quantified following nigericin exposure in the presence and absence of siRNAs targeting caspase-3/7 (Fig. 5(F, G)). Compared to non-coding controls, caspase-3/7 suppression led to an accumulation of cells in Stage 2 (“Ring-of-fire”) (Supplemental Figure 8G–H) and a significant concomitant decrease in the proportion of cells in Stage 3 (“Pyroptotic bodies”) (Fig. 5(F), Supplemental Figure 8G–H). The failure of cells to form pyroptotic bodies in the absence of caspase-3/7 suggested an active role for caspase-3/7 (or their substrates) in pyroptotic body formation (Supplemental Figure 8H). Inhibition of caspase-3 or -7 individually did not reproduce this phenotype, implying functional redundancy between the two pro-apoptotic caspases (Supplemental Figure 8G). Importantly, GSDMD expression levels were not reduced in the context of caspase-3/7 suppression, ruling out the possibility that caspase-3/7 affected pyroptotic body formation through disruption of GSDMD expression (Supplemental Figure 8I). Of note, ROCK1, a prototypic substrate of caspases-3 and -7, is pivotal to the formation of apoptotic bodies through cytoskeletal reorganization during apoptosis [14]; as shown earlier, cleaved ROCK1 was significantly increased in microglia exposed to nigericin or ATP as well as staurosporine (*p* < 0.0001) (Fig. 4(D,G)), implying ROCK1 might be involved in the formation of pyroptotic bodies.

### Caspase-3/7 accumulate in the nucleus and contribute to nuclear condensation during pyroptosis

**Fig. 6:**
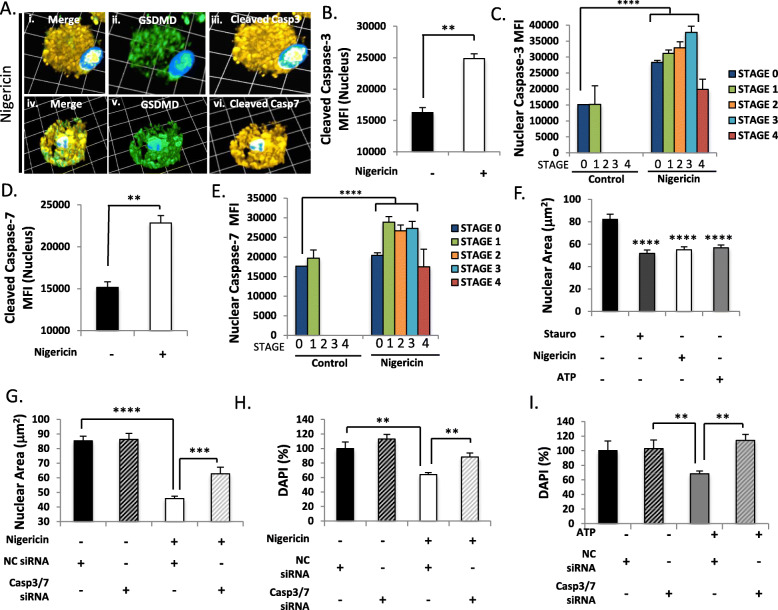
Activated caspase-3/7 accumulate in the nucleus and promote nuclear shrinkage during pyroptosis. (**a**) Human microglia were exposed to nigericin (5.0 μM, 4 h), fixed, and immunolabelled for GSDMD (green) and either cleaved caspase-3 or -7 (amber). Images shown are representative 3-dimensional z-stacks incorporating 15 XY planes over a vertical distance of 4–6 μm. One square unit represents 10.28 μm. (**b**–**e**) Mean fluorescence intensity (MFI) of cleaved caspase-3 (**b**, **c**) and caspase-7 (**d**, **e**) in the nucleus was assessed in unexposed (*n* = 50) and nigericin-exposed (minimum *n* = 150) microglia and categorized by pyroptotic stages. Data shown represent mean nuclear cleaved caspase-3/7 MFI ± SEM for a representative human donor. Results were independently verified in microglia from 2 to 3 separate human donors (**p < 0.001, Student’s *t* test). (F) Microglia were exposed to staurosporine (5 μM, 4 h), nigericin (5 μM, 4 h) or ATP (100 μM, 24 h), fixed and DAPI-stained. Cross-sectional area (μm^2^) of DAPI-stained nuclei were measured (*n* = 10–20 cells per condition). Data shown represent mean nuclear area ± SEM for a representative human donor. Data were tested for significance using one-way ANOVA with Dunnett’s test for multiple comparisons (****p < 0.0001). (G) Microglia were transfected with either universal non-coding siRNA (NC siRNA) or a cocktail of different siRNAs targeting caspases-3 and -7 (Casp-3/7 siRNA) prior to nigericin exposure (5 μM, 24 h). Cells were fixed, DAPI stained to label nuclei, and assessed by confocal microscopy. Cross-sectional area (μm^2^) of DAPI-stained nuclei were measured in unexposed (*n* = 20) and nigericin-exposed (minimum *n* = 50) microglia. Data shown represent mean nuclear area ± SEM for a representative human donor (***p < 0.001, ****p < 0.0001, analyzed using one-way ANOVA with Dunnett’s test for multiple comparisons). (H, I) Cells were transfected with either universal non-targeting siRNA (NT siRNA) or a cocktail of different siRNAs targeting caspase-3 and -7 (Casp-3/7 siRNA) prior to (H) nigericin (5 μM, 24 h) or (I) ATP (100 μM, 24 h) exposure. Total DAPI levels within each population were assessed fluorometrically by microplate reader, with a minimum of *n* = 8 technical replicates per condition (**p < 0.01, ***p < 0.001, ****p < 0.0001, analyzed using one-way ANOVA with Dunnett’s test for multiple comparisons).

Following nigericin exposure, cleaved caspase-3/7 immunoreactivity was abundant in the nucleus (Fig. 6(A)), significantly increasing in the nuclei of cells undergoing pyroptosis at all time points tested (*p* < 0.0001) (Fig. 6(A–E), Supplemental Figure 9A, C). Stage-dependent analyses verified this increase in nuclear cleaved caspase-3/7 MFI as cells progressed through pyroptosis (Fig. 6(C, E) and Supplemental Figure 9B, D). Concomitantly, DAPI^+^ nuclear cross-sectional area declined significantly between Stages 3 and 4 (Supplemental Figure 9E, F), verifying previous observations of nuclear condensation during end-stage pyroptosis [15, 41].

This finding prompted the examination of whether caspase-3/7 might mediate nuclear condensation during pyroptotic cell death. The DAPI^+^ nuclear cross-sectional area was measured in microglia exposed to pro-pyroptotic (nigericin or ATP) or pro-apoptotic (staurosporine) stimuli. These studies demonstrated a comparable and significant decrease in nuclear cross-sectional area for all conditions tested (Fig. 6(F)) (*p* < 0.0001). The effect of exposing microglia to nigericin was then investigated in the presence of siRNAs targeting caspase-3/7 or universal non-coding control siRNA (Fig. 6(G)). Nigericin exposure caused a significant decrease in nuclear cross-sectional area, which was partially prevented by caspase-3/7 inhibition (*p* < 0.001) (Fig. 6(G)). To extend these observations, total DAPI signal in the population was also measured as a readout of the relative number and size of nuclei within cells. Overall DAPI signal decreased significantly during pyroptosis following exposure to either nigericin or ATP (Fig. 6(H, I)), which was rescued by caspase-3/7 inhibition (*p* < 0.01).

### Caspase-3 cleavage is inhibited by VX-765

**Fig. 7:**
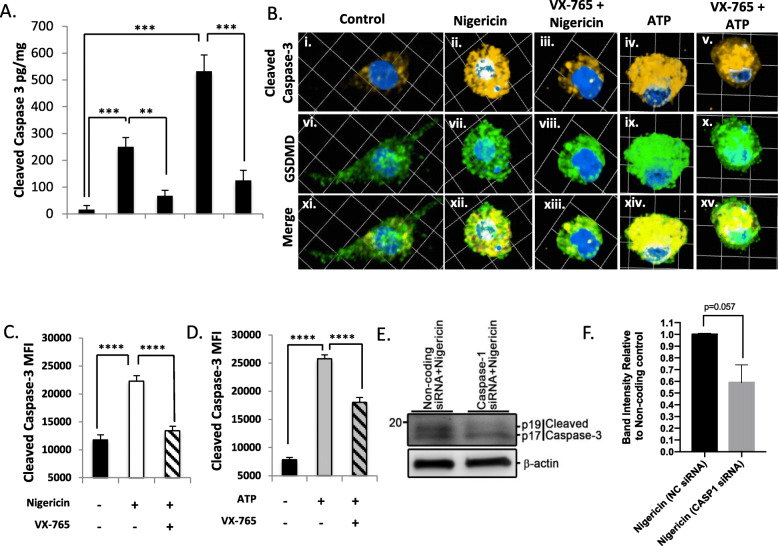
Caspase-3 activation occurs downstream of canonical caspase-1 inflammasome activation. (**a**) Microglia were pre-treated with VX-765 (50 μM, 4 h) and then exposed to pyroptotic stimuli ATP (100.0 μM, 24 h) or nigericin (5.0 μM, 4 h), from which lysates were harvested and cleaved caspase-3 levels assessed by ELISA. Data shown represent mean cleaved caspase-3 levels ± SEM (*n* = 6 technical replicates) from a representative human donor. Results were confirmed in microglia from 3 to 5 donors. (**b**) Microglia were exposed as in (**a**) and immunolabelled for cleaved caspase-3 p17/p19 (amber) and GSDMD (green). Images represent three-dimensional z-stacks incorporating 15 XY planes over a vertical distance of 4–6 μm. (**c**, **d**) Mean fluorescence intensity (MFI) of cleaved caspase-3 was assessed in control microglia (minimum *n* = 20) and nigericin- or ATP-exposed microglia ± VX-765 (minimum *n* = 100). Data shown represent mean cleaved caspase-3 MFI ± SEM for a representative human donor. Results were independently verified in microglia from 2 to 3 separate donors. (**e**, **f**) Microglia were transfected with either universal non-coding siRNA (NC siRNA) or a cocktail of three different siRNAs targeting caspase-1, exposed to nigericin, and cell lysates immunoblotted for caspase-3 as indicated. Data shown are mean protein band intensities ± SEM (*n* = 2 biological replicates; one-tailed Student’s *t* test) normalized to beta-actin, expressed relative to the untreated NC control. P17 and p19 bands were quantified collectively to quantify the total reduction in cleaved caspase-3 peptide

To test if inflammasome activation was required for caspase-3 cleavage during pyroptosis in human microglia, cells were pre-treated with the caspase-1 inhibitor VX-765 [37, 38]. VX-765 pre-treatment reduced cleaved caspase-3 in cell lysates following nigericin or ATP exposure, as determined by a high-sensitivity cleaved caspase-3 ELISA, suggesting caspase-1 activation was upstream of caspase-3 cleavage (Fig. 7(A)). To assess whether intracellular cleaved caspase-3 MFI was likewise decreased by pre-treatment with VX-765, confocal microscopy was performed. Notably, VX-765 did not inhibit caspase-3 cleavage during staurosporine-induced apoptosis (Supplemental Figure 10A, B). In contrast, the increase in cleaved caspase-3 MFI following nigericin or ATP exposure was significantly reduced by caspase-1 inhibition (*p* < 0.0001) (Fig. 7(B–D)). Intracellular caspase-7 immunoreactivity was also was significantly reduced with VX-765 treatment (*p* < 0.001) (Supplemental Figure 4A, B). To validate these results, siRNA-mediated knockdown of caspase-1 was performed, and caspase-3 cleavage assessed by immunoblot. These results demonstrated that overall caspase-3 cleavage was substantially inhibited when caspase-1 was knocked down (Fig. 7(E, F)). Of interest, the p19 caspase-3 fragment (which often predominates during caspase-1-mediated cleavage of caspase-3 [28, 30]) was virtually abolished by caspase-1 knockdown, while the p17 caspase-3 fragment (which predominates during caspase-8/9-mediated cleavage of caspase-3 [28, 30]) persisted. It is likely that the p17 fragment observed herein was derived from a cell stress response to the transfection reagent, which was not caspase-1-dependent. Collectively, these data highlighted that caspase-3/7 activation occurred downstream of inflammasome activation and could be inhibited with VX-765 during pyroptosis.

### Cleaved caspase-3 is co-expressed with GSDMD in microglia/macrophages during EAE

**Fig. 8:**
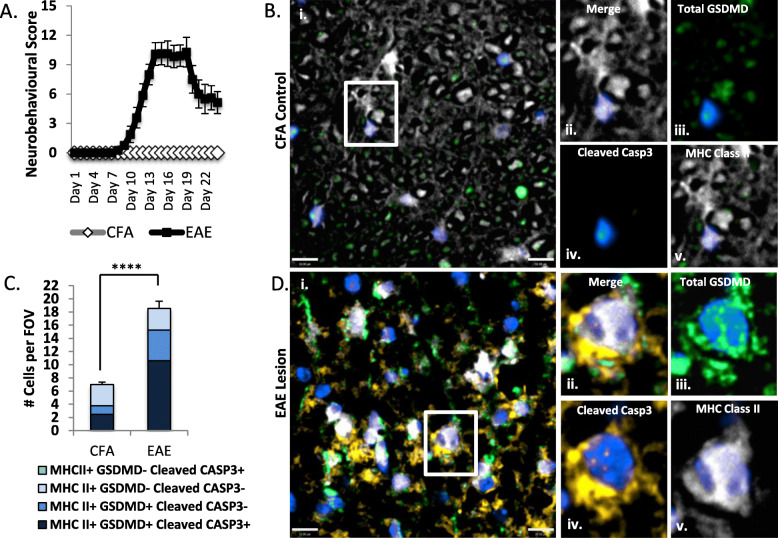
Macrophages/microglia co-express GSDMD and cleaved caspase-3 in the CNS during EAE: EAE was induced in C57/Bl6 female animals with CFA exposure as a control (*n* = 10 per group). Animals were sacrificed at 24 days post-induction of disease for morphological analyses. (A) Neurobehavioural outcomes in EAE and CFA-exposed animals, measured using a 15-point severity scale. (B–D) Spinal cord tissue sections from CFA-exposed (B) and EAE mice (D) were immunolabelled for cleaved caspase-3 (amber), total GSDMD (green), and MHC class II (white) and imaged using confocal microscopy (representative images are shown). (C) Each MHC class II^+^ cell was categorized as single-immunopositive, double-immunopositive, or double-immunonegative for total GSDMD and cleaved caspase-3 based on mean fluorescence intensity (MFI) using a threshold of 3X background fluorescence. A total of *n* = 224 MHC class II^+^ cells from 32 unique fields of view (FOV) were categorized from *n* = 4 CFA-exposed animals. A total of *n* = 630 MHC class II^+^ cells from 34 unique FOV derived from *n* = 4 EAE animals were categorized. Distribution of cells within each of the categories was significantly different between CFA control white matter and EAE white matter lesions by Chi-square test (****p < 0.0001). Scale bar = 10 μm

Having implicated caspase-3/7 activation in human microglia/macrophages undergoing pyroptosis, we sought to determine whether this observation was preserved during neuroinflammation in a prototypic murine model of multiple sclerosis. We previously identified glial pyroptosis in the C57/Bl6 EAE model and demonstrated that pharmacological inhibition of caspase-1 using VX-765 was neuroprotective in EAE [9]. This model involves a monophasic disease course in which neurobehavioral signs become evident 8–10 days following disease induction, with disease severity peaking approximately one week later; this is followed by a partial recovery in the chronic disease phase (Fig. 8(A)). Using this model, we demonstrated that MHC class II immunopositive microglia/macrophages in spinal cord white matter from CFA-only control mice displayed minimal GSDMD (green) or cleaved caspase-3 (amber) immunoreactivity (Fig. 8(B)). In contrast, EAE lesions contained abundant microglia/macrophages that were double immunopositive for both cleaved caspase-3 (amber) and GSDMD (green) (Fig. 8(C, D)). Both the average number of GSDMD^+^ cells per field of view (FoV) and the average number of double immunopositive cells increased significantly during EAE compared to CFA-exposed animals (*p* < 0.0001), as did overall microglia/macrophage numbers (*p* < 0.0001). While CFA controls had on average 2.4 ± 0.27 cleaved caspase-3/GSDMD double immunopositive cells per FoV, EAE animals had an average of 10.6 ± 0.8 double immunopositive cells in spinal cord lesions per FOV (Fig. 8(C)). Concurrently, the proportion of double immunopositive cells increased from 35% in CFA-exposed controls to 57% in active EAE lesions (Fig. 8(C)). Overall, 82.4% of all microglia/macrophages within the lesion were GSDMD immunopositive. Cleaved caspase-3 single immunopositive cells were not identified in microglia/macrophage populations in spinal cords from either EAE or CFA-exposed animals, indicating that apoptosis was unlikely to contribute to myeloid cell death in this model.

## Discussion

Pyroptosis has recently been identified as a major contributor to pathogenesis in multiple neurological conditions, including traumatic brain injury [18, 19], sepsis-associated encephalopathy [20, 21], Alzheimer’s disease [22], and MS [9, 23]. In the context of MS and its animal model EAE, myeloid cell death is often under-appreciated relative to other neuropathological features of the disease (such as immune cell infiltration, oligodendrocyte loss, and neuronal injury); nonetheless, a robust historical precedent exists for myeloid cell death in MS/EAE, where it was hypothesized to result from activity-induced cell death [42] or iron overload [43]. TUNEL-positive macrophage and microglial populations are detectable during EAE [42, 44], though this was historically interpreted as evidence of apoptosis. More recent reports have provided ample evidence for CNS myeloid cell pyroptosis during EAE based upon caspase-1/11 staining, GSDMD expression/activation, and propidium iodide staining [9, 23]. These observations collectively make MS/EAE an appropriate model for investigating the mechanisms that drive pyroptosis of macrophages/microglia during neuroinflammation.

**Fig. 9:**
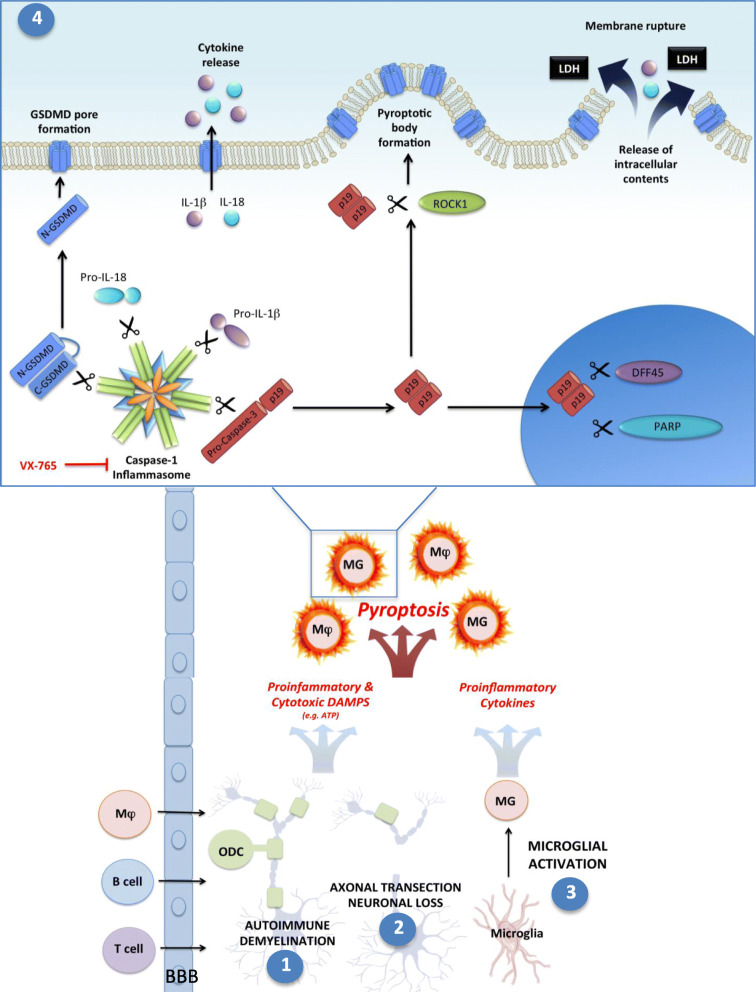
Activated caspases-3 and -7 contribute to pyroptosis in macrophage/microglia in multiple sclerosis. MS neuropathogenesis is driven by converging disease processes that create a proinflammatory microenvironment, including infiltration of peripheral immune cells, demyelination (1), axonal loss and neuronal cell death (2), and microglial activation (3). Widespread cell death releases proinflammatory DAMPs and alarmins (e.g., extracellular ATP), which trigger pyroptosis in macrophages (Mφ)/microglia (MG). Pyroptosis is lytic and highly proinflammatory, creating a positive feedback loop to drive further pyroptotic cell death. Within the pyroptotic cell (4), inflammasome activation leads to the cleavage and activation of proinflammatory cytokines (IL-1β and IL-18) and the cytotoxic pore-forming protein GSDMD, and as shown in this manuscript, procaspase-3. While activated GSDMD translocates to the plasma membrane to form pores, cleaved caspase-3 traffics to multiple cellular compartments wherein it cleaves several substrates that promote pyroptosis. In the nucleus, caspase-3 and -7 substrates DFF45 and PARP are cleaved and activated. ROCK1 is cleaved and activated in the cytoplasm and appears to concentrate around the cell periphery and in pyroptotic bodies, suggesting a putative role in pyroptotic body formation that mimics its role in apoptotic body formation during apoptosis. Collectively, the proteolytic network engaged by caspase-3/7 during pyroptosis activates parallel processes that facilitate cellular demolition, pyroptotic body formation, and membrane lysis during pyroptosis

The present manuscript provides the first evidence to our knowledge that the apoptotic executioners, caspase-3 and -7, contribute to GSDMD-mediated pyroptosis, thus providing unprecedented insight into the molecular mechanisms governing pyroptosis in the CNS (Fig. 9).

Using MS and EAE as prototypic models of neuroinflammation, we first verified our earlier studies that had demonstrated GSDMD immunoreactivity in Iba-1 immunopositive macrophages/microglia; although there were subtle differences in the EAE model (the disease course in the current study was more severe and tissue was harvested during the chronic rather than peak stage of disease), the overall percentage of GSDMD immunopositive macrophages/microglia in spinal cord lesions was consistent (~ 80%) in both studies (Fig. 8(C)) [9]. We observed that active caspase-3 was highly expressed in pyroptotic CNS macrophages/microglia in MS and EAE lesions (Figs. 1 and 8), providing compelling evidence that cleaved caspase-3 is *not* a unique molecular marker of apoptosis, as is widely assumed in the context of CNS disease. This observation was also validated in vitro, wherein activated caspase-3/7 and their cleaved substrates (DFF45, ROCK1, and PARP) were found to accumulate during microglial pyroptosis (Fig. 4). In a functional screen of different caspase and gasdermin family members, inhibition of caspase-1, -3, -7, or GSDMD (but not caspase-8, -9, or GSDME) significantly reduced pyroptosis-associated cell lysis, and inhibition of both caspase-3/7 virtually abrogated cell lysis (Fig. 3). These experiments provided compelling evidence for a functional contribution of caspase-3/7 to pyroptosis. When caspase-3/7 were blocked, GSDMD accumulated at the plasma membrane but the formation of pyroptotic bodies was markedly reduced (Fig. 5), thereby offering a mechanism by which plasma membrane rupture might be abrogated by caspase-3/7 inhibition. Importantly, targeting caspase-1 either genetically or pharmacologically reduced caspase-3/7 activation, suggesting that caspase-1 is upstream of caspase-3/7 activation and offering a therapeutic opportunity for caspase-3/7 inhibition. Taken together, our findings support the hypothesis that pyroptotic stimuli can activate a network of caspases, including caspase-1, -3, and -7, all of which participate in pyroptotic cell death of CNS macrophages/microglia. Of note, MHC class II^+^ macrophages and microglia were not distinguished from each other in our in vivo studies; as both populations are important in MS pathogenesis, further studies could delineate the effects of caspase-3 and GSDMD activation in individual brain macrophage populations.

The hypothesis that caspase-3/7 mediate a complex intracellular demolition process that occurs in parallel to GSDMD activation during microglial pyroptosis counters the view that GSDMD-mediated membrane rupture is the singular defining molecular feature of pyroptotic cell death. Interestingly, several groups have demonstrated subcellular changes, such as loss of mitochondrial function and cell motility, prior to GSDMD-mediated membrane rupture [45, 46], suggesting a broader disruption of intracellular functions reminiscent of that observed in apoptosis. The present study supports the concept that pyroptosis engages a network of proteases, which ultimately facilitate GSDMD-mediated membrane rupture in CNS macrophages/microglia.

Multiple lines of evidence lend credence to the notion that caspase-1, not caspase-8 or -9, mediate caspase-3/7 activation during microglial pyroptosis. First, cell-free proteolysis assays have previously demonstrated that purified caspase-1 can directly cleave and activate the apoptotic executioner caspases-3/7 [27]. The caspase-1 cleavage event predominantly generates a bioactive p19 caspase-3 fragment rather than the p17 caspase-3 fragment that predominates during caspase-8- or caspase-9-mediated cleavage events [28, 30]. This observation is consistent with the size of the active caspase-3 fragments detected following treatment with nigericin, wherein the p19 fragment was abolished upon siRNA-mediated caspase-1 inhibition (Fig. 7(E, F)). Secondly, activity-dependent fluorescent probes (Fig. 4(H)) failed to provide evidence for caspase-8 or -9 activation during pyroptosis, while confirming that caspase-3/7 activity was abundant. Thirdly, the LDH screen (Fig. 3(D, E)) did not reveal any reduction in cell lysis accompanying caspase-8 or -9 inhibition, unlike inhibition of caspases-1, -3, and -7, which prevented pyroptosis-associated cell lysis. Lastly, inhibition of caspase-1 reduced caspase-3/7 activation during pyroptosis but not apoptosis (Fig. 7, Supplemental Figure 4), suggesting a role for caspase-1 upstream of caspase-3/7 activation. Thus, our results support the hypothesis that caspase-3/7 were activated at least in part by caspase-1 during pyroptosis, and caspase-3/7 in turn facilitated pyroptosis by activating a proteolytic network that facilitated cellular demolition.

While the present study reveals a previously unrecognized contribution of caspase-3/7 to GSDMD-mediated pyroptosis in CNS microglia/macrophages, other groups have reported that caspase-3 cleaves and activates the related protein GSDME, a phenomenon that has been best characterized in the context of cancer [7, 47]. Our results are conspicuously different from these latter studies for several reasons: (i) GSDME inhibition had no effect on pyroptosis in the present studies (Fig. 3), indicating that the caspase-3/GSDME axis was not a determinant of pyroptosis in this system, and (ii) our observations do not support a molecular cascade in which caspase-3 directly cleaves and activates GSDMD, as is proposed for GSDME. Unlike GSDME, GSDMD contains a caspase-3 cleavage site that generates a permanently inactive p45 fragment in response to apoptotic stimuli [48]. To address the question of why caspase-3 does not cleave and inactivate GSDMD in our system, we performed immunofluorescence at early time points and found that active p30 N-GSDMD was detectable by immunofluorescence prior to active caspase-3 detection (Supplemental Figure 10C); thus, GSDMD cleavage and activation likely precedes the emergence of active caspase-3 and in doing so precludes the generation of the inactive p45 GSDMD peptide.

Within the context of pyroptosis, the range of caspase-3/7 substrates with functional significance to pyroptosis remains uncertain. Given the accumulation of activated caspase-3/7 in multiple cellular compartments (including the nucleus, cytoplasm, and pyroptotic bodies), it is plausible that different caspase-3/7 substrates differentially contribute to the morphological features of pyroptosis, including nuclear disintegration and pyroptotic body formation (Fig. 9). In apoptosis, the caspase-3/7 substrate ROCK1 initiates membrane-associated apoptotic body formation through regulation of the actin-myosin cytoskeleton, resulting in the separation of the plasma membrane from the cytoskeleton [14]. In this study, we demonstrated that cleaved (i.e., active) ROCK1 accumulated during pyroptosis at levels comparable to apoptosis (Fig. 4(D, G)) in a caspase-3/7-dependent manner (Supplemental Figure 5E, F) and appeared enriched within pyroptotic bodies (Fig. 4(D), Supplemental Figure 5E). These observations imply a potential role for ROCK1 in pyroptotic body formation and suggest that in the absence of caspase-3/7, ROCK1 cannot be cleaved and activated, thus preventing the cytoskeletal modifications required to form pyroptotic bodies, which are in turn required for cell lysis. Rupture of pyroptotic bodies represents the point-of-no-return in pyroptosis and is typically thought to be driven by passive osmotic swelling. However, in the present study, caspase-3/7 inhibition prevented pyroptotic body formation (Fig. 5(F, G)), representing the first evidence for a direct contribution by caspase-3/7 to pyroptotic body formation. This suggests that pyroptotic body formation is not simply a stochastic response to changing osmotic gradients, but rather an active process that can be modulated, as reported for the formation of apoptotic bodies in apoptosis [49]. Activated ROCK1 might initiate the cytoskeletal modifications required to enable plasma membrane detachment from the cytoskeleton, as occurs in apoptosis [14]; once detached, the plasma membrane may passively swell and burst as a result of the changes in osmotic pressure that emerge as a consequence of GSDMD pores concentrated within the pyroptotic body. While apoptotic bodies are generally non-immunogenic, have intact membranes, and are loaded with cellular contents such as DNA/organelles for uptake by phagocytes [49], it is plausible that pyroptotic bodies might contain cargo with proinflammatory effects such as cytokines, proteases, and other alarmins to amplify the inflammatory response upon rupture of the pyroptotic body. The detection of robust IL-1β immunoreactivity within microglial pyroptotic bodies supports this concept [9].

The present model, in which primary human microglia are utilized to study pyroptosis, has several strengths that warrant recognition: (i) brain macrophages/microglia have been shown to undergo pyroptosis in vivo in multiple disease systems, highlighting the clinical relevance of our system; (ii) using non-transformed primary microglia circumvents the problem of investigating transformed cells with aberrant cell death pathways; and finally (iii) studying primary human microglia from diverse donors precludes species-specific artifacts associated with murine systems and clonal effects from genetically uniform experimental systems.

As previously reported [9], primary human microglia under these culture conditions do not require a separate priming step to undergo nigericin- or ATP-induced pyroptosis; this is likely due to the constitutive expression of inflammasome components (such as NLRP3, NLRP1, and caspase-1) under resting conditions in microglia compared to donor-matched astrocytes or neurons [33]. Likewise, both GSDMD and IL-1β are detectable at the protein level in resting microglia [9], removing the requirement for a separate priming step.

It remains to be determined whether the involvement of caspase-3/7 in pyroptosis is a unique evolutionary adaptation of microglia/macrophages to the CNS microenvironment, or whether this phenomenon is conserved within other cell populations. It is important to consider that the mechanisms underlying RCD, including pyroptosis, are not universally shared among all model systems; these variations depend on species (human versus mouse), tissue type (CNS versus periphery), stimulus (PAMPs versus DAMPs), and cell type (primary cells versus transformed cancer cells), all which can potentially affect the molecular mechanism of cell death. As such, the role for caspase-3/7 in facilitating pyroptosis in human microglia may not apply to all cell types; likewise, a dispensable role for caspase-3/7 (individually or in combination) in some models of pyroptosis does not preclude their importance in human microglia.

## Conclusion

In summary, we have identified a novel caspase-1-dependent function for caspase-3/7 in pyroptosis of macrophages/microglia in the context of neuroinflammation. These findings provide new mechanistic insights into pyroptotic cell death in the CNS, and may offer therapeutic opportunities for MS and other neuroinflammatory diseases in which pyroptosis participates in pathogenesis.

## Supplementary information

**Additional file 1: Table S1.** Neuropathological and Demographic Characteristics of Autopsy Tissue Donors. **Figure S1.** (A) To validate the specificity of the antibody for cleaved GSDMD, THP-1 GSDMD knockout (KO) cells were exposed to nigericin (5.0 μM, 4hrs), alongside THP-1 mock KO cells and immunoblotted. Only cleaved (31 kDa) not full-length (53 kDa) GSDMD was detected in lysates following nigericin treatment. **Figure S2.** (A) Autopsied tissue sections from non-MS white matter or MS patient white matter lesions were stained for Luxol Fast Blue (LFB), CD68, or CD3 together with H&E labeling and imaged by light microscopy to assess demyelination, macrophage/microglial activation and T cell infiltration (scale bar = 100μm). (B) Autopsied brain tissue sections from non-MS white matter or progressive MS patient white matter lesions were immunolabelled for MHC Class II and the number of positive cells quantified and analyzed using Student’s t–test (**** p<0.0001). Data shown are mean number of MHC Class II+ cells per FOV+/- SEM, n=40 FOVs for nonMS; n=88 FOVs for MS lesions (C) Each MHC Class II^+^ cell from Fig. 1 A-B was categorized as single-immunopositive, double-immunopositive, or double-immunonegative for total GSDMD and cleaved GSDMD based on mean fluorescence intensity (MFI), using a threshold of 3X background fluorescence. Data shown are mean number of MHC Class II+ cells per FOV+/- SEM. A total of n=78 MHC Class II^+^ cells from 10 unique fields of view (FOV) derived from the neuropathologically normal nonMS control were categorized; n= 295 MHC Class II^+^ cells from 30 unique FOV derived from two MS patients were categorized. The difference in absolute numbers of double positive cells was tested by Student’s t-test (**** p<0.0001). (D) Each MHC Class II^+^ cell from Fig. 1 D-G was categorized as single-immunopositive, double-immunopositive, or double-immunonegative for cleaved caspase-3 and GSDMD based on MFI using a threshold of 3X background fluorescence. Data shown are mean number of MHC Class II+ cells per FOV+/- SEM. A total of n=209 MHC Class II^+^ cells from 29 unique fields of view (FOV) derived from two neuropathologically normal nonMS controls were categorized; n= 223 MHC Class II^+^ cells from 25 unique FOV derived from the normal-appearing white matter (NAWM) of two MS patients were also categorized, along with n=554 MHC Class II^+^ cells from 58 unique fields of view (FOV) within MS lesions. The difference in absolute numbers of double positive cells was tested by one-way ANOVA (*** *p*<0.001, **** *p*<0.0001). **Figure S3.** (A-G) To validate the siRNAs utilized in the LDH screen, microglia were transfected with either universal non-coding siRNA (NC siRNA) or a cocktail of three different siRNAs targeting caspase-3 and -7 (Casp-3/7 siRNA) (A-C); GSDMD (D,E); GSDME (F,G); caspase-1 (H,I); caspase-4 (J,K); Caspase-9 (L,M); or caspase-8 (N,O) prior to nigericin exposure (5μM, 4hrs). Lysates were harvested at the indicated timepoints post-exposure and immunoblotted for the indicated proteins and β-actin. Data shown are protein band intensities normalized to beta-actin, expressed relative to the untreated NC control. **Figure S4.** (A) Human microglia were exposed to nigericin (5μM, 4hrs) or ATP (100μm, 24hrs) +/- VX-765 (50μM, 4hr pre-treatment), fixed, and immunolabelled for cleaved caspase-7 (amber). (B) MFI of cleaved caspase-7 was assessed in a minimum of n=30 cells per condition. Data were tested for significance using 1-way ANOVA with Dunnett’s test for multiple comparisons (***p<0.001). **Figure S5.** (A,B) Microglia were transfected with either universal non-coding siRNA (NC siRNA) or a combination of different siRNAs targeting caspase-3 and -7 (Casp-3/7 siRNA) prior to nigericin exposure (5μM, 4hrs). Lysates were harvested and immunoblotted for cleaved PARP. Data shown are protein band intensities normalized to beta-actin, expressed relative to the untreated non-coding control. (C-F) Microglia were transfected with either universal non-coding siRNA (NC siRNA) or a cocktail of different siRNAs targeting caspase-3 and -7 (Casp-3/7 siRNA) prior to nigericin exposure (5μM, 4hrs). Cells were fixed, immunolabelled for cleaved DFF45 (C; cyan) or ROCK1 (E; pink) and assessed by confocal microscopy (nuclei in blue, indicated by white arrows). Representative 2-dimensional images shown. (D) Nuclear mean fluorescence intensity (MFI) of cleaved DFF45 was assessed in unexposed (n=20) and nigericin-exposed (minimum n=50) cells. Microglia in Stage 0 were excluded from analysis. (F) Mean fluorescence intensity (MFI) of cleaved ROCK1 was assessed in unexposed and nigericin-exposed cells (minimum n=20 per condition). Microglia in Stage 0 were excluded from analysis. Data shown represent mean MFI +/- SEM for a representative human donor and were analyzed using 1-way ANOVA with Dunnett’s test for multiple comparisons (****p<0.0001). All results were independently recapitulated using microglia derived from 2-3 separate human donors. **Figure S6.** Microglia were exposed to nigericin (5μM, 4hrs) or exogenous human IL-18 or IL-1β (25 ng/mL) for 24hrs, fixed and immunolabelled for cleaved caspase-3 (amber) and GSDMD (green). Representative images are shown. (B) Proportion of microglia at each stage of pyroptosis is shown.(C, D) Loss of cell membrane integrity following exposure to human IL-1β (C) or IL-18 (D) was assessed using an LDH activity assay. IL-1β- and IL-18-exposed microglia displayed no change in supernatant LDH activity (Student’s t-test, NS = non-significant). (E) Cleaved caspase-3 MFI was assessed for a minimum of 25 cells from (A) per condition. Data shown represent mean MFI +/- SEM for a representative human donor (****p<0.0001, NS = non-significant). **Figure S7.** (A) Schematic showing stages of pyroptosis and predicted action of VX-765. (B) Human microglia were exposed to nigericin (5μM, 4hrs) +/- VX-765 (50μM, 4 hr pre-treatment), fixed, and immunolabelled for GSDMD. Each cell was classified by stage (0-5) of pyroptosis. Proportion of microglia at each stage of pyroptosis was calculated using a minimum of n=20 cells for controls and n=100 cells per treatment condition. Distribution of cells across the stages was significantly different in VX-765-pre-treated microglia using Chi-square test. Results are shown for a representative human donor and were replicated in microglia derived from 2-4 independent donors (**p<0.01). (C-E) Microglia were pre-treated with VX-765 (50μM, 4hrs) and then exposed to nigericin (5.0 μM, 4hrs), fixed, and immunolabelled for cleaved GSDMD (red) and total GSDMD (green), and visualized by confocal microscopy. Mean fluorescence intensity (MFI) of total GSDMD (D) and cleaved GSDMD (E) was assessed. Data shown represent mean MFI +/- SEM for a representative human donor. Results were independently verified in microglia from 2-3 separate donors. **Figure S8.** (A-F) Human microglia were exposed to nigericin (5μM, 1hr, 4hr, 24hr), fixed, immunolabelled for cleaved caspase-3, cleaved caspase-7, or GSDMD and visualized using confocal microscopy. Cleaved caspase-3 (A,B), caspase-7 (C,D), or GSDMD MFI (E,F) was quantified at each time point for the microglia classified in stages, using n=60 control microglia and a minimum of n=100 nigericin-exposed microglia per time point. Data shown represent MFI +/- SEM for a representative human donor (*** p<0.001, analyzed using 1-way ANOVA with Dunnett’s test for multiple comparisons). (G) Microglia were transfected with either universal non-targeting siRNA (NT siRNA) or a cocktail of siRNAs targeting either caspase-3 or-7 or both prior to nigericin exposure (5μM, 24hrs). Cells were fixed, immunolabelled for GSDMD and cleaved caspase-3, and categorized by stage of pyroptosis (Stages 0-5) using confocal microscopy. Distribution of the cells across the stages was assessed using a Chi-squared test (** p<0.01). (H) Stages of pyroptosis with predicted effect of caspase-3/7 inhibition. (I) Mean fluorescence intensity (MFI) of GSDMD was assessed (n=20-30 cells per condition treated as in (H). Data shown represent mean MFI +/- SEM for a representative human donor and were analyzed using 1-way ANOVA with Dunnett’s test for multiple comparisons. All results were independently confirmed in microglia derived from 2-3 separate human donors. **Figure S9.** Human microglia were exposed to nigericin (5μM, 1hr, 4hr, 24hr), fixed, immunolabelled for cleaved caspase-3 or cleaved caspase-7 and visualized using confocal microscopy. Mean fluorescence intensity (MFI) of cleaved caspase-3 (A,B) or cleaved caspase-7 (C,D) in the nucleus was assessed for a minimum of n=60 control microglia and n=80 microglia per time point post-nigericin exposure. (B,D) Each cell was classified by stage (0-4) of pyroptosis and nuclear MFI expressed separately for each stage of pyroptosis at the indicated timepoints. Data shown represent mean MFI +/- SEM for a representative human donor. Results were independently recapitulated in microglia from 2 separate human donors. Data were tested for significance using 1-way ANOVA with Dunnett’s test for multiple comparisons (**** p<0.0001). (E,F) Cross-sectional area of each nucleus (defined by DAPI staining) was measured and cross-sectional area expressed separately for each stage of pyroptosis at the indicated time points for a minimum of n=150 microglia per time point. Data shown represent mean area +/- SEM for a representative human donor. Data were tested for significance using 1-way ANOVA with Dunnett’s test for multiple comparisons (**p<0.01, **** p<0.0001). **Figure S10.** (A) Human microglia were exposed to staurosporine (1μM) +/- VX-765 (50μM, 4hr pre-treatment), fixed, immunolabelled for cleaved caspase-3 (amber) and GSDMD (green) and visualized using confocal microscopy. (B) MFI was assessed in cell bodies for a minimum of n=40 microglia per group. Data shown represent mean MFI +/- SEM for a representative human donor. NS = not significant. (C) Human microglia were exposed to nigericin (5μM) for 15mins, fixed and immunolabelled for total GSDMD, cleaved caspase-3, or cleaved GSDMD. Data shown represent mean MFI +/- SEM for a representative human donor (***p<0.001).

## Data Availability

Any additional data not included herein is available from the corresponding author upon reasonable request.

## Abbreviations

ATP: Adenosine tri-phosphate
CFA: Complete Freud’s adjuvant
CNS: Central nervous system
DAMP: Damage-associated molecular pattern
EAE: Experimental autoimmune encephalomyelitis
FOV: Field of view
GSDMD: Gasdermin D (executioner of pyroptosis)
LDH: Lactate dehydrogenase
MFI: Mean fluorescence intensity
MHC: Major histocompatibility complex
MS: Multiple sclerosis
NAWM: Normal-appearing white matter
PAMP: Pathogen-associated molecular pattern
RCD: Regulated cell death
