# Supplementary material for: Activation of the executioner caspases-3 and -7 promotes microglial pyroptosis in models of multiple sclerosis
Source: J Neuroinflammation. 2020 Aug 29;17:253. doi: 10.1186/s12974-020-01902-5 (PMC7456507; doi:10.1186/s12974-020-01902-5)
Supplement: Supplementary file 1 — Additional file 1: Table S1. Neuropathological and Demographic Characteristics of Autopsy Tissue Donors. Figure S1. (A) To validate the specificity of the antibody for cleaved GSDMD, THP-1 GSDMD knockout (KO) cells were exposed to nigericin (5.0 μM, 4hrs), alongside THP-1 mock KO cells and immunoblotted. Only cleaved (31 kDa) not full-length (53 kDa) GSDMD was detected in lysates following nigericin treatment. Figure S2. (A) Autopsied tissue sections from non-MS white matter or MS patient white matter lesions were stained for Luxol Fast Blue (LFB), CD68, or CD3 together with H&E labeling and imaged by light microscopy to assess demyelination, macrophage/microglial activation and T cell infiltration (scale bar = 100μm). (B) Autopsied brain tissue sections from non-MS white matter or progressive MS patient white matter lesions were immunolabelled for MHC Class II and the number of positive cells quantified and analyzed using Student’s t–test (**** p<0.0001). Data shown are mean number of MHC Class II+ cells per FOV+/- SEM, n=40 FOVs for nonMS; n=88 FOVs for MS lesions (C) Each MHC Class II+ cell from Fig. 1 A-B was categorized as single-immunopositive, double-immunopositive, or double-immunonegative for total GSDMD and cleaved GSDMD based on mean fluorescence intensity (MFI), using a threshold of 3X background fluorescence. Data shown are mean number of MHC Class II+ cells per FOV+/- SEM. A total of n=78 MHC Class II+ cells from 10 unique fields of view (FOV) derived from the neuropathologically normal nonMS control were categorized; n= 295 MHC Class II+ cells from 30 unique FOV derived from two MS patients were categorized. The difference in absolute numbers of double positive cells was tested by Student’s t-test (**** p<0.0001). (D) Each MHC Class II+ cell from Fig. 1 D-G was categorized as single-immunopositive, double-immunopositive, or double-immunonegative for cleaved caspase-3 and GSDMD based on MFI using a threshold of 3X background fluorescence. Data shown ar [file 12974_2020_1902_MOESM1_ESM.pptx]

## Slide 1
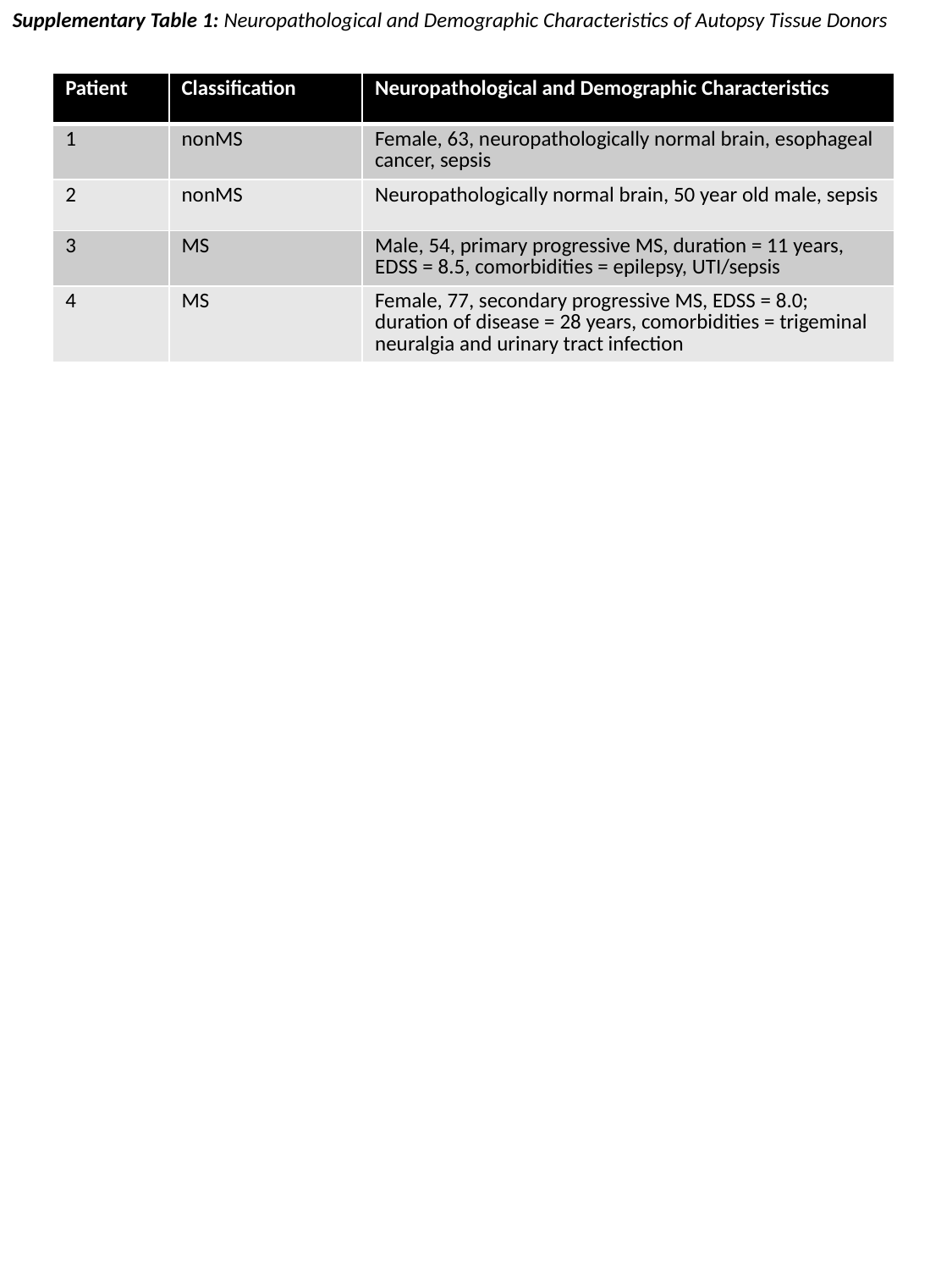

Supplementary Table 1: Neuropathological and Demographic Characteristics of Autopsy Tissue Donors
| Patient | Classification | Neuropathological and Demographic Characteristics |
| --- | --- | --- |
| 1 | nonMS | Female, 63, neuropathologically normal brain, esophageal cancer, sepsis |
| 2 | nonMS | Neuropathologically normal brain, 50 year old male, sepsis |
| 3 | MS | Male, 54, primary progressive MS, duration = 11 years, EDSS = 8.5, comorbidities = epilepsy, UTI/sepsis |
| 4 | MS | Female, 77, secondary progressive MS, EDSS = 8.0; duration of disease = 28 years, comorbidities = trigeminal neuralgia and urinary tract infection |

## Slide 2
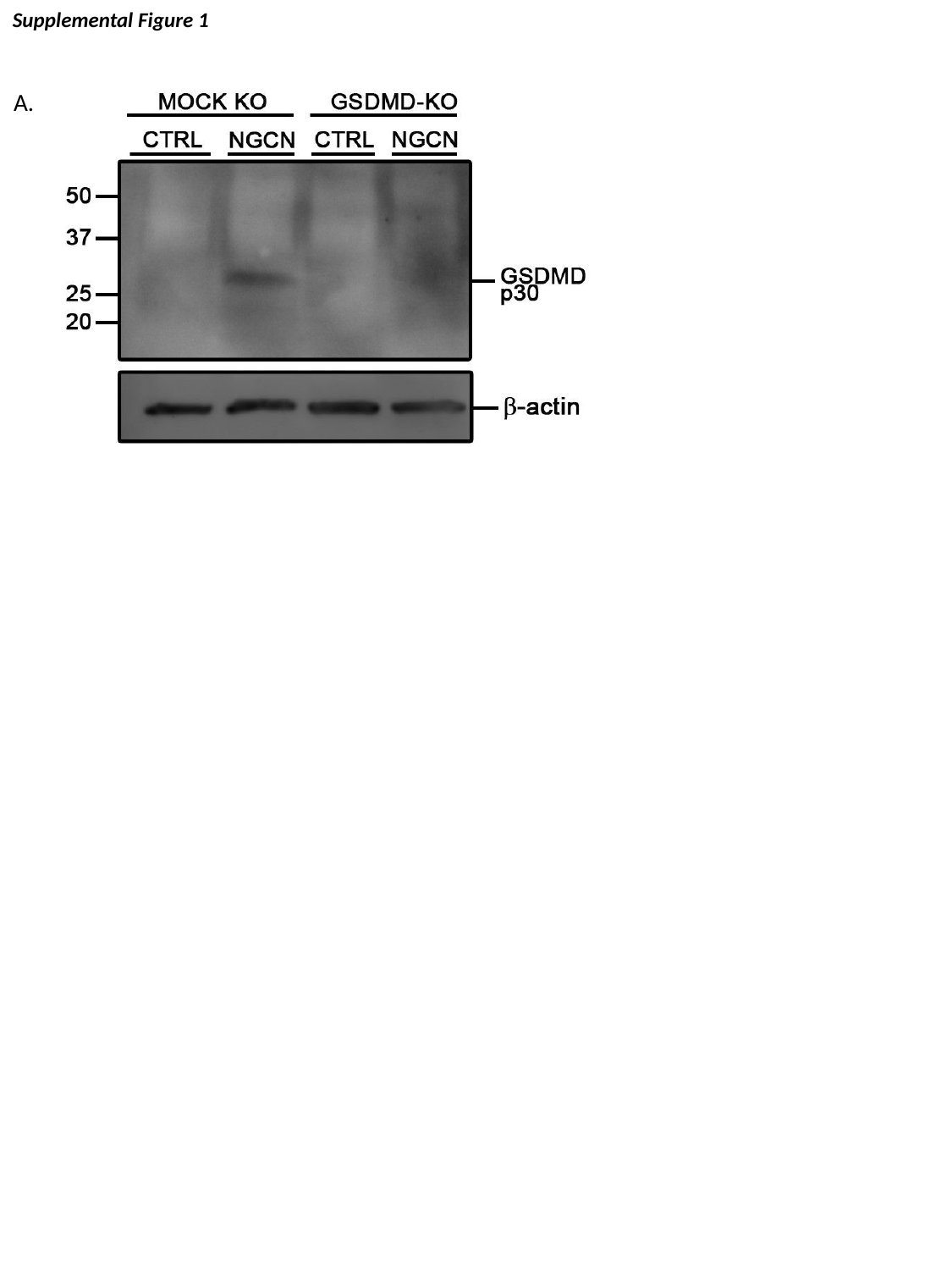

Supplemental Figure 1
A.

## Slide 3
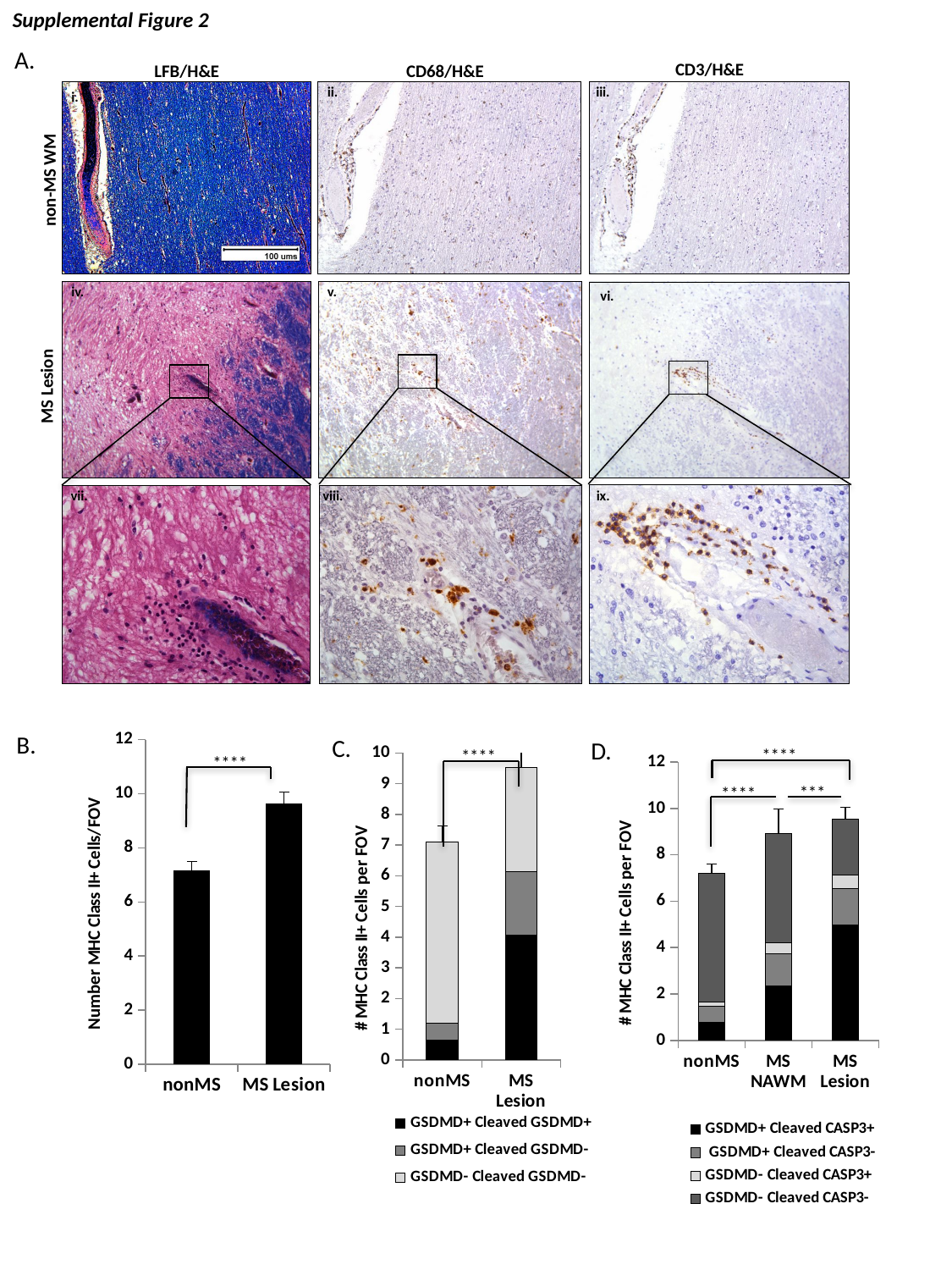

Supplemental Figure 2
A.
CD3/H&E
LFB/H&E
CD68/H&E
i.
ii.
iii.
i.
non-MS WM
iv.
v.
vi.
 MS Lesion
vii.
viii.
ix.
B.
### Chart
| Category | Mean |
|---|---|
| nonMS | 7.175 |
| MS Lesion | 9.647727272727273 |
### Chart
| Category | GSDMD+ Cleaved GSDMD+ | GSDMD+ Cleaved GSDMD- | GSDMD- Cleaved GSDMD- |
|---|---|---|---|
| nonMS | 0.636363636363636 | 0.545454545454545 | 5.90909090909091 |
| MS Lesion | 4.064516129032251 | 2.064516129032258 | 3.387096774193548 |C.
D.
### Chart
| Category | GSDMD+ Cleaved CASP3+ | GSDMD+ Cleaved CASP3- | GSDMD- Cleaved CASP3+ | GSDMD- Cleaved CASP3- |
|---|---|---|---|---|
| nonMS | 0.793103448275862 | 0.689655172413793 | 0.172413793103448 | 5.551724137931034 |
| MS NAWM | 2.36 | 1.36 | 0.48 | 4.72 |
| MS Lesion | 4.965517241379306 | 1.568965517241379 | 0.603448275862069 | 2.413793103448276 |****
****
****
***
****

## Slide 4
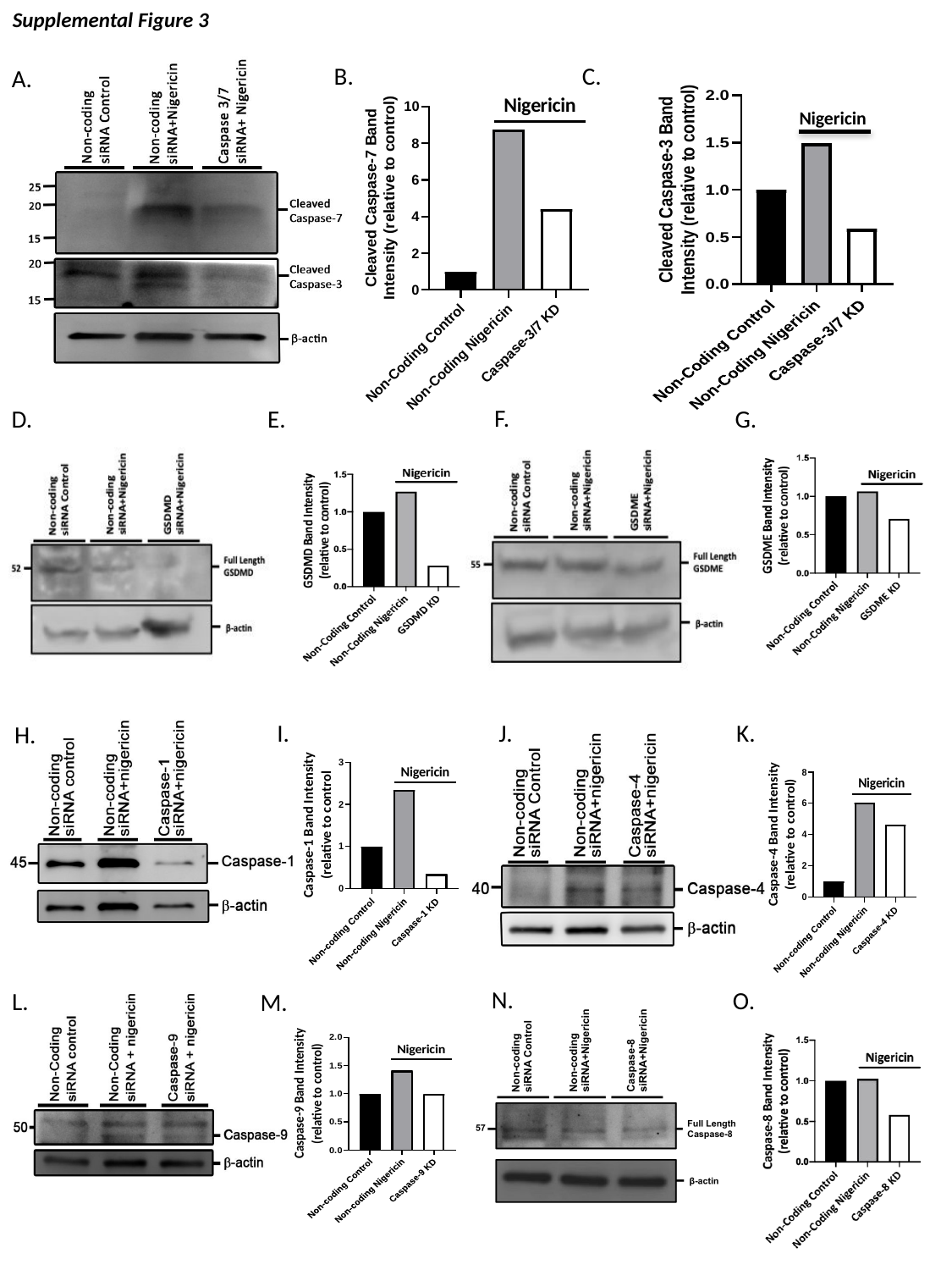

Supplemental Figure 3
A.
B.
C.
B.
C.
A.
 Nigericin
F.
D.
E.
G.
J.
I.
K.
H.
N.
O.
L.
M.

## Slide 5
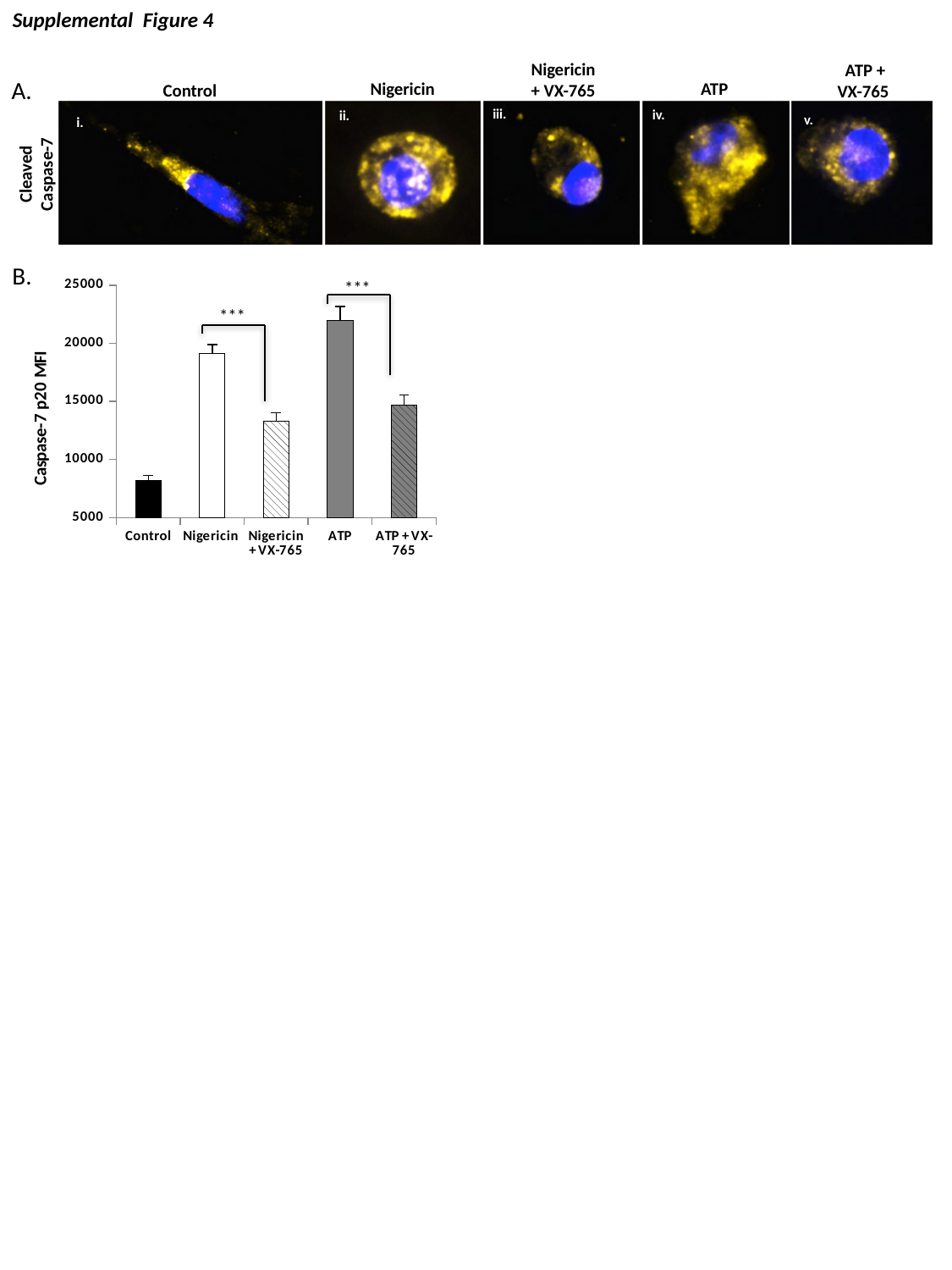

Supplemental Figure 4
Nigericin + VX-765
ATP + VX-765
A.
Nigericin
ATP
Control
iii.
iv.
ii.
iii.
v.
i.
Cleaved Caspase-7
vi.
B.
***
### Chart
| Category | Caspase-7 MFI Mean |
|---|---|
| Control | 8205.58064516129 |
| Nigericin | 19098.54545454546 |
| Nigericin + VX-765 | 13254.13725490196 |
| ATP | 21926.54237288135 |
| ATP + VX-765 | 14696.82352941176 |***

## Slide 6
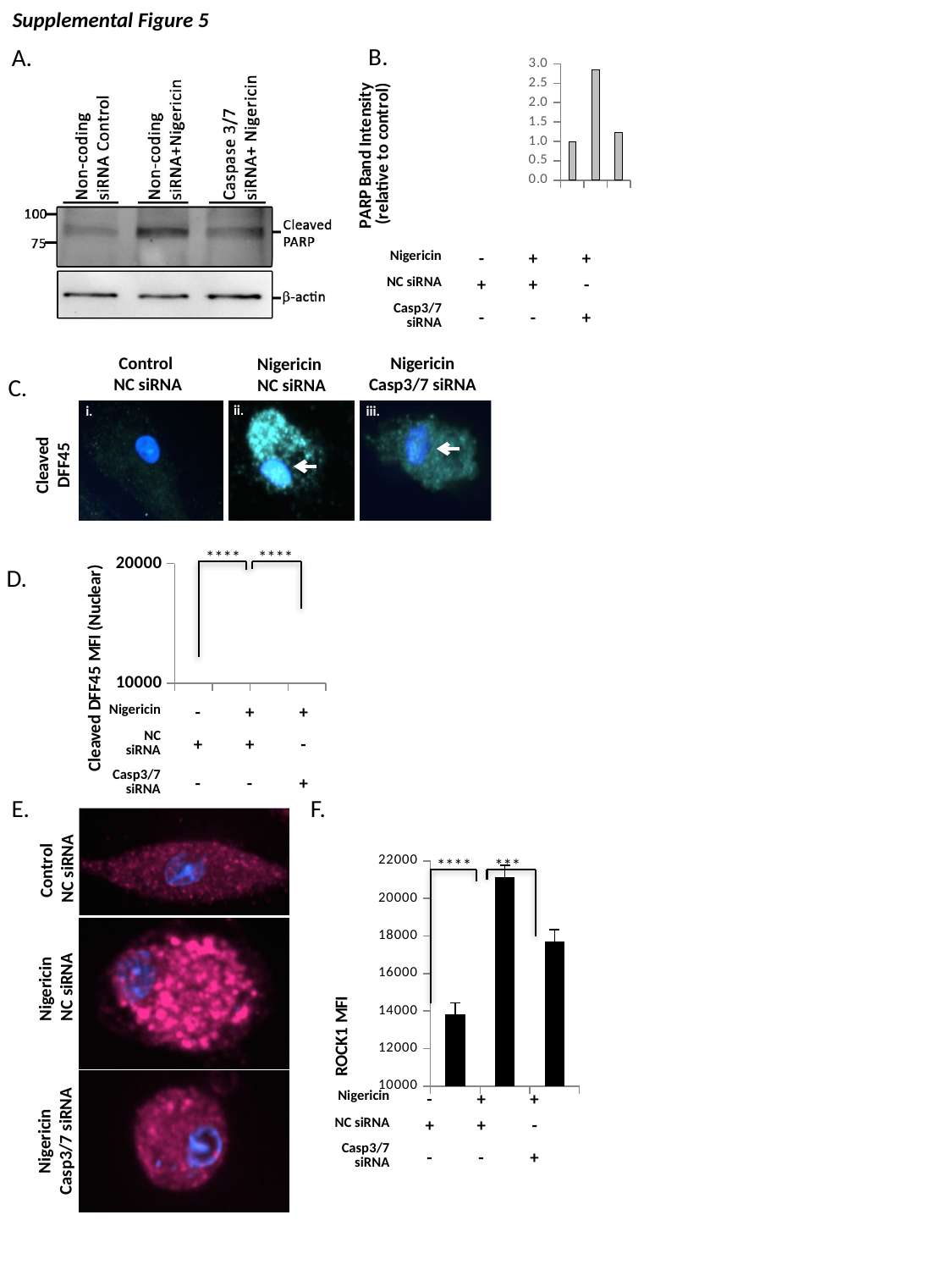

Supplemental Figure 5
B.
A.
### Chart
| Category | Cleaved PARP |
|---|---|
| Non-coding Control | 1.0 |
| Non-coding+Nigericin | 2.85 |
| C3/C7 siRNA +nigericin | 1.24 || Nigericin | - | + | + |
| --- | --- | --- | --- |
| NC siRNA | + | + | - |
| Casp3/7 siRNA | - | - | + |
Control
NC siRNA
Nigericin Casp3/7 siRNA
Nigericin
NC siRNA
ii.
iii.
i.
Cleaved DFF45
C.
### Chart
| Category | Mean |
|---|---|
| Control NC | 11393.11764705882 |
| Nigericin NC | 33578.1403508772 |
| Nigericin C3C7 | 23899.29032258064 |****
****
D.
| Nigericin | - | + | + |
| --- | --- | --- | --- |
| NC siRNA | + | + | - |
| Casp3/7 siRNA | - | - | + |
F.
E.
Control
NC siRNA
### Chart
| Category | Mean |
|---|---|
| Control NC siRNA | 13844.64516129032 |
| Nigericin NC siRNA | 21136.14925373134 |
| Nigericin C3/C7 siRNA | 17695.44736842105 |****
***
Nigericin
NC siRNA
| Nigericin | - | + | + |
| --- | --- | --- | --- |
| NC siRNA | + | + | - |
| Casp3/7 siRNA | - | - | + |
Nigericin
Casp3/7 siRNA

## Slide 7
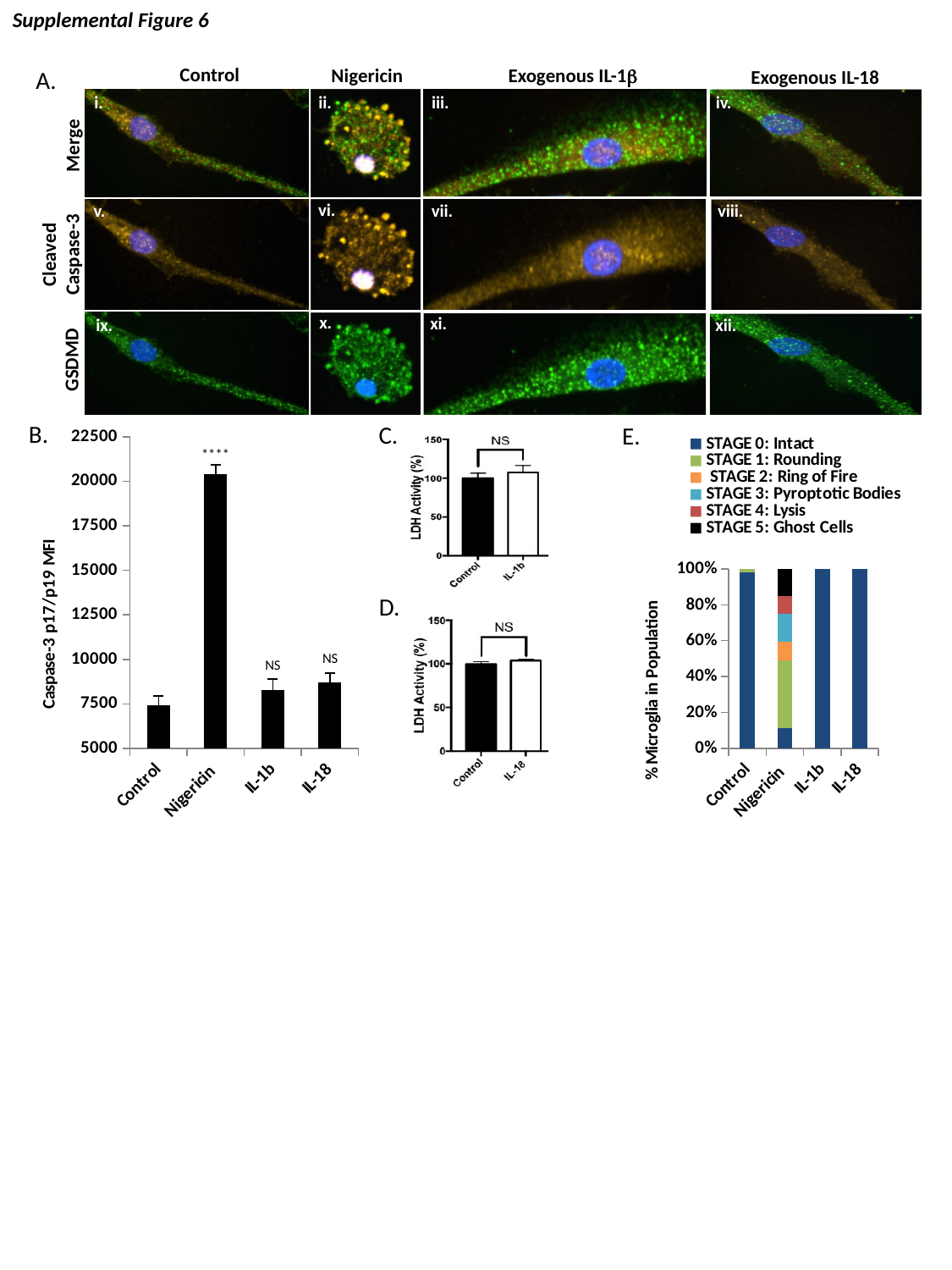

Supplemental Figure 6
Control
Nigericin
Exogenous IL-1b
A.
Exogenous IL-18
i.
ii.
iii.
iv.
Merge
vi.
v.
vii.
viii.
Cleaved Caspase-3
x.
xi.
ix.
xii.
GSDMD
### Chart
| Category | |
|---|---|
| Control | 7431.16666666667 |
| Nigericin | 20386.6338028169 |
| IL-1b | 8285.961538461523 |
| IL-18 | 8722.44 |B.
C.
E.
### Chart
| Category | STAGE 0: Intact | STAGE 1: Rounding | STAGE 2: Ring of Fire | STAGE 3: Pyroptotic Bodies | STAGE 4: Lysis | STAGE 5: Ghost Cells |
|---|---|---|---|---|---|---|
| Control | 47.0 | 1.0 | 0.0 | 0.0 | 0.0 | 0.0 |
| Nigericin | 19.0 | 63.0 | 17.0 | 26.0 | 17.0 | 25.0 |
| IL-1b | 25.0 | 0.0 | 0.0 | 0.0 | 0.0 | 0.0 |
| IL-18 | 25.0 | 0.0 | 0.0 | 0.0 | 0.0 | 0.0 |
****
D.
NS
NS

## Slide 8
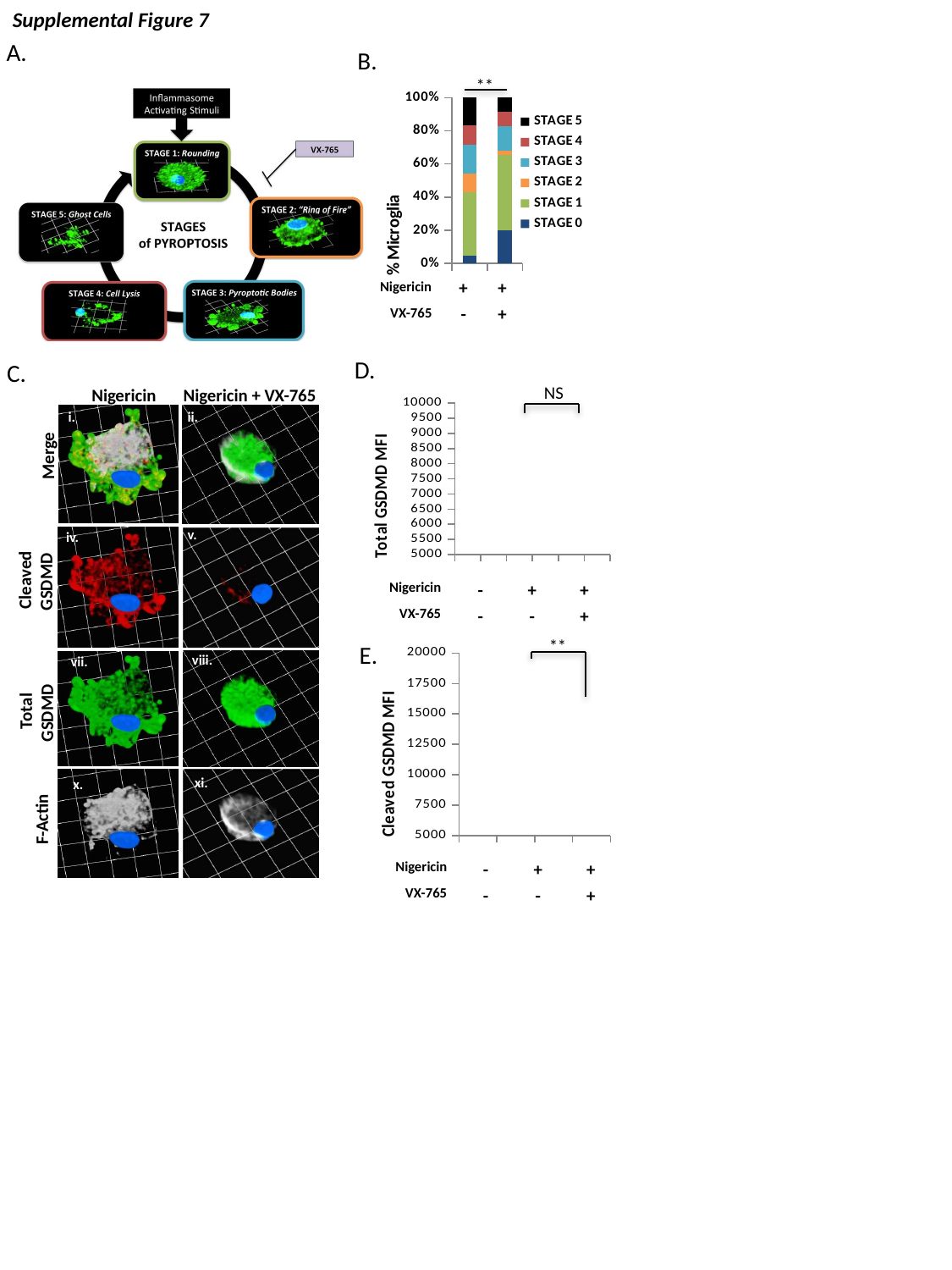

Supplemental Figure 7
A.
B.
**
### Chart
| Category | STAGE 0 | STAGE 1 | STAGE 2 | STAGE 3 | STAGE 4 | STAGE 5 |
|---|---|---|---|---|---|---|
| Nigericin | 5.0 | 42.0 | 12.0 | 19.0 | 13.0 | 18.0 |
| Nigericin + VX-765 | 21.0 | 47.0 | 3.0 | 15.0 | 9.0 | 9.0 || Nigericin | + | + |
| --- | --- | --- |
| VX-765 | - | + |
D.
C.
Nigericin + VX-765
Nigericin
i.
ii.
 Merge
v.
iv.
Cleaved GSDMD
viii.
vii.
 Total GSDMD
xi.
x.
F-Actin
NS
### Chart
| Category | Mean | SE |
|---|---|---|
| Control | 12503.42105263158 | 1352.863473844391 |
| Nigericin | 31558.52941176471 | 1484.100228701878 |
| Nigericin + VX-765 | 28107.41176470588 | 1257.348971911838 || Nigericin | - | + | + |
| --- | --- | --- | --- |
| VX-765 | - | - | + |
**
E.
### Chart
| Category | Mean |
|---|---|
| Control | 8525.368421052632 |
| Nigericin | 18123.9705882353 |
| Nigericin + VX-765 | 14986.25490196078 || Nigericin | - | + | + |
| --- | --- | --- | --- |
| VX-765 | - | - | + |

## Slide 9
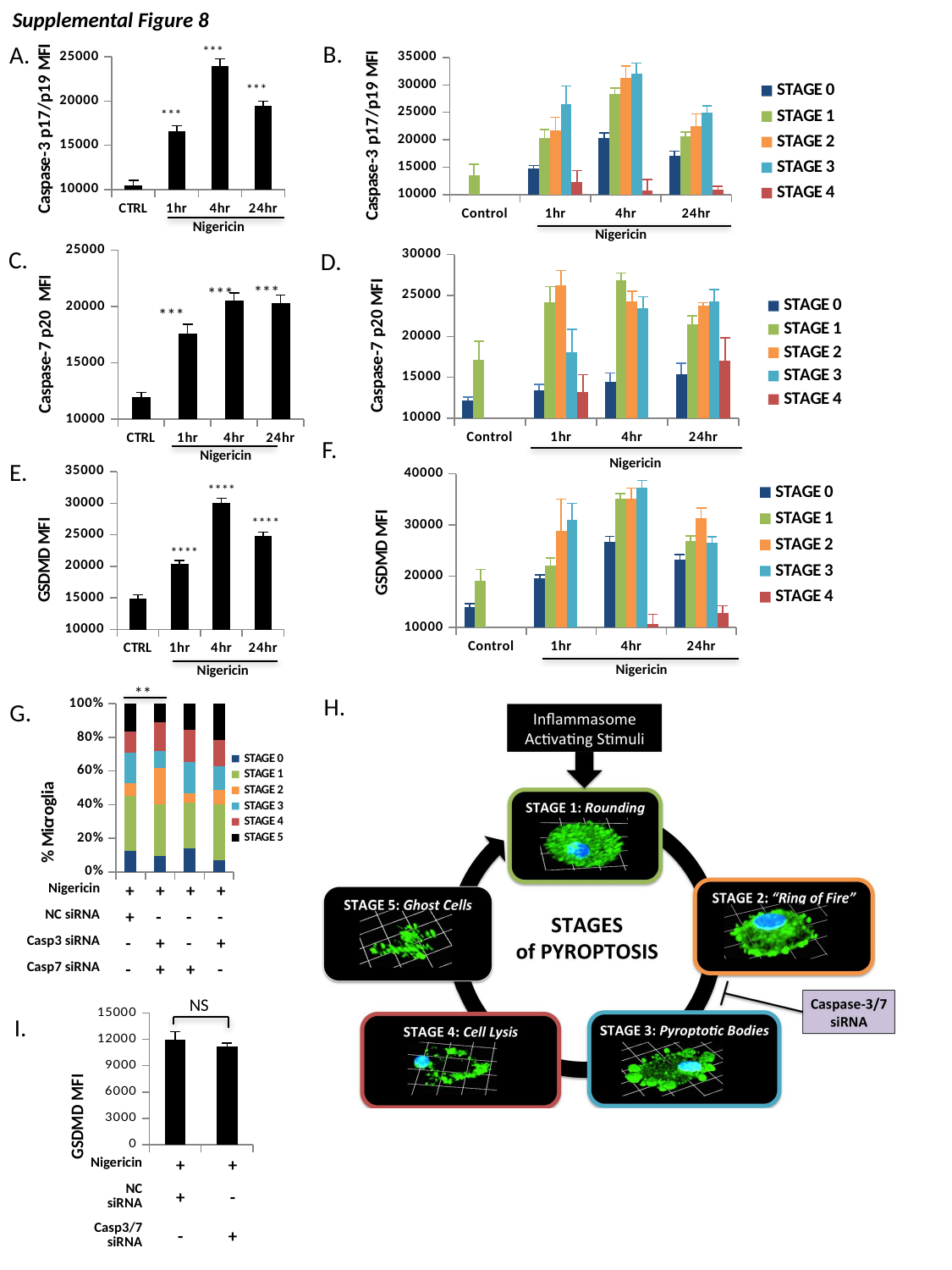

Supplemental Figure 8
B.
A.
***
### Chart
| Category | CASP3 |
|---|---|
| CTRL | 10477.7 |
| 1hr | 16576.61157024789 |
| 4hr | 23948.55102040815 |
| 24hr | 19420.08333333333 |***
***
Nigericin
### Chart
| Category | STAGE 0 | STAGE 1 | STAGE 2 | STAGE 3 | STAGE 4 |
|---|---|---|---|---|---|
| Control | 9763.58490566033 | 13574.16666666667 | None | None | None |
| 1hr | 14717.18072289157 | 20402.28571428571 | 21736.28571428571 | 26448.5 | 12310.33333333333 |
| 4hr | 20315.31818181818 | 28396.61764705883 | 31241.83333333331 | 32053.14285714286 | 10825.14285714286 |
| 24hr | 17110.25641025641 | 20572.3432835821 | 22519.57142857143 | 24919.19047619048 | 10855.0 |Nigericin
C.
### Chart
| Category | CASP7 |
|---|---|
| CTRL | 11977.1195652174 |
| 1hr | 17604.64423076923 |
| 4hr | 20487.82835820895 |
| 24hr | 20301.69512195122 |***
***
***
### Chart
| Category | STAGE 0 | STAGE 1 | STAGE 2 | STAGE 3 | STAGE 4 |
|---|---|---|---|---|---|
| Control | 12142.55555555555 | 17073.85714285714 | None | None | None |
| 1hr | 13389.66666666667 | 24220.77272727273 | 26225.54545454546 | 18093.11111111111 | 13208.2 |
| 4hr | 14404.6603773585 | 26908.19444444445 | 24296.83333333331 | 23453.55555555555 | 9494.66666666666 |
| 24hr | 15363.4 | 21522.68571428572 | 23723.5 | 24264.23529411765 | 17029.36363636364 |D.
F.
### Chart
| Category | GSDMD |
|---|---|
| CTRL | 14809.61666666667 |
| 1hr | 20332.94214876033 |
| 4hr | 29912.67676767669 |
| 24hr | 24809.59615384615 |
### Chart
| Category | STAGE 0 | STAGE 1 | STAGE 2 | STAGE 3 | STAGE 4 |
|---|---|---|---|---|---|
| Control | 14006.37735849057 | 19147.66666666667 | None | None | None |
| 1hr | 19637.59036144575 | 22110.66666666667 | 28930.6 | 31036.75 | 9201.0 |
| 4hr | 26803.4888888889 | 35239.85294117646 | 35089.0 | 37344.85714285714 | 10636.875 |
| 24hr | 23320.33333333331 | 26975.70149253732 | 31370.5 | 26629.40740740741 | 12856.42857142857 |****
****
****
Nigericin
Nigericin
Nigericin
Nigericin
E.
**
H.
G.
### Chart
| Category | STAGE 0 | STAGE 1 | STAGE 2 | STAGE 3 | STAGE 4 | STAGE 5 |
|---|---|---|---|---|---|---|
| Non-coding | 13.0 | 34.0 | 8.0 | 19.0 | 13.0 | 17.0 |
| C3 + C7 siRNA | 13.0 | 42.0 | 29.0 | 14.0 | 23.0 | 15.0 |
| C7 siRNA | 8.0 | 16.0 | 3.0 | 11.0 | 11.0 | 9.0 |
| C3 siRNA | 5.0 | 23.0 | 6.0 | 10.0 | 11.0 | 15.0 |
| Nigericin | + | + | + | + |
| --- | --- | --- | --- | --- |
| NC siRNA | + | - | - | - |
| Casp3 siRNA | - | + | - | + |
| Casp7 siRNA | - | + | + | - |
NS
I.
### Chart
| Category | GSDMD |
|---|---|
| Nigericin NC siRNA | 11989.71590909091 |
| Nigericin CASP3/7 siRNA | 11156.02150537634 || Nigericin | + | + |
| --- | --- | --- |
| NC siRNA | + | - |
| Casp3/7 siRNA | - | + |

## Slide 10
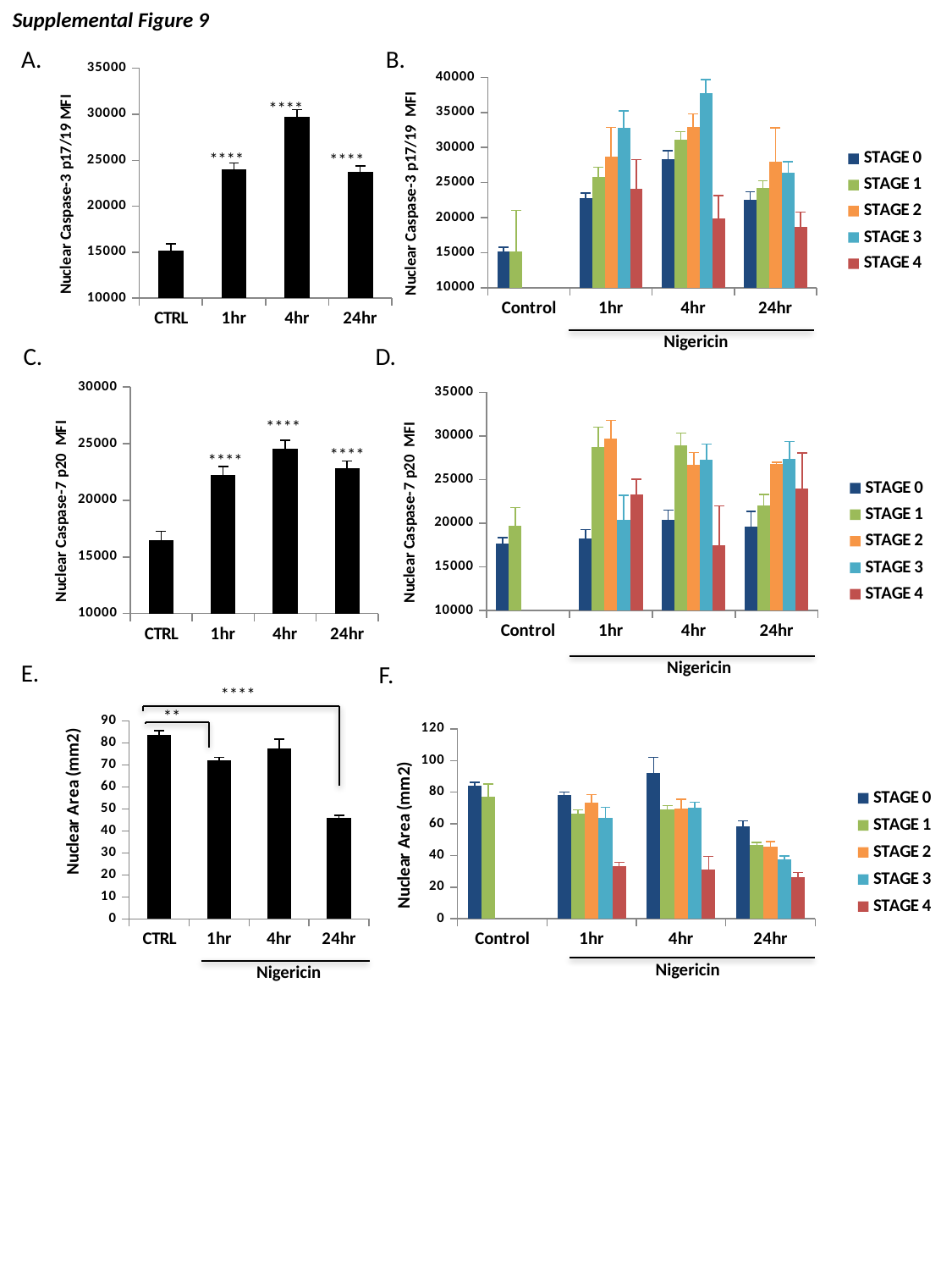

Supplemental Figure 9
A.
B.
### Chart
| Category | NUCLEAR |
|---|---|
| CTRL | 15144.6779661017 |
| 1hr | 24001.47863247863 |
| 4hr | 29725.09375 |
| 24hr | 23753.41290322581 |
### Chart
| Category | STAGE 0 | STAGE 1 | STAGE 2 | STAGE 3 | STAGE 4 |
|---|---|---|---|---|---|
| Control | 15138.90566037736 | 15195.66666666667 | None | None | None |
| 1hr | 22725.1851851852 | 25778.95238095238 | 28721.28571428571 | 32756.75 | 24109.0 |
| 4hr | 28350.4761904762 | 31161.18181818182 | 32901.33333333334 | 37743.14285714286 | 19837.57142857143 |
| 24hr | 22583.97435897436 | 24236.16666666667 | 27986.66666666667 | 26416.96296296296 | 18683.07142857143 |****
****
****
Nigericin
C.
D.
### Chart
| Category | STAGE 0 | STAGE 1 | STAGE 2 | STAGE 3 | STAGE 4 |
|---|---|---|---|---|---|
| Control | 17648.55223880589 | 19681.83333333331 | None | None | None |
| 1hr | 18192.8392857143 | 28693.68181818182 | 29743.09090909091 | 20367.77777777778 | 23262.0 |
| 4hr | 20415.88461538462 | 28897.35294117645 | 26654.5 | 27292.85185185185 | 17437.66666666667 |
| 24hr | 19624.13333333334 | 22006.33333333331 | 26789.5 | 27403.73333333333 | 23962.8888888889 |
### Chart
| Category | NUCLEAR |
|---|---|
| CTRL | 16476.81818181818 |
| 1hr | 22266.8640776699 |
| 4hr | 24541.15384615385 |
| 24hr | 22835.64102564103 |****
****
****
Nigericin
E.
F.
****
### Chart
| Category | |
|---|---|
| CTRL | 83.80132450331126 |
| 1hr | 72.02714932126698 |
| 4hr | 77.3695652173913 |
| 24hr | 45.85355648535509 |**
### Chart
| Category | STAGE 0 | STAGE 1 | STAGE 2 | STAGE 3 | STAGE 4 |
|---|---|---|---|---|---|
| Control | 84.33333333333285 | 77.36363636363635 | None | None | None |
| 1hr | 78.088888888887 | 66.5476190476191 | 73.38888888888654 | 63.58333333333334 | 33.4 |
| 4hr | 92.17525773195734 | 69.11428571428571 | 69.5 | 70.08571428571427 | 31.2 |
| 24hr | 58.2962962962963 | 46.40594059405941 | 45.625 | 37.58139534883721 | 26.45454545454545 |Nigericin
Nigericin

## Slide 11
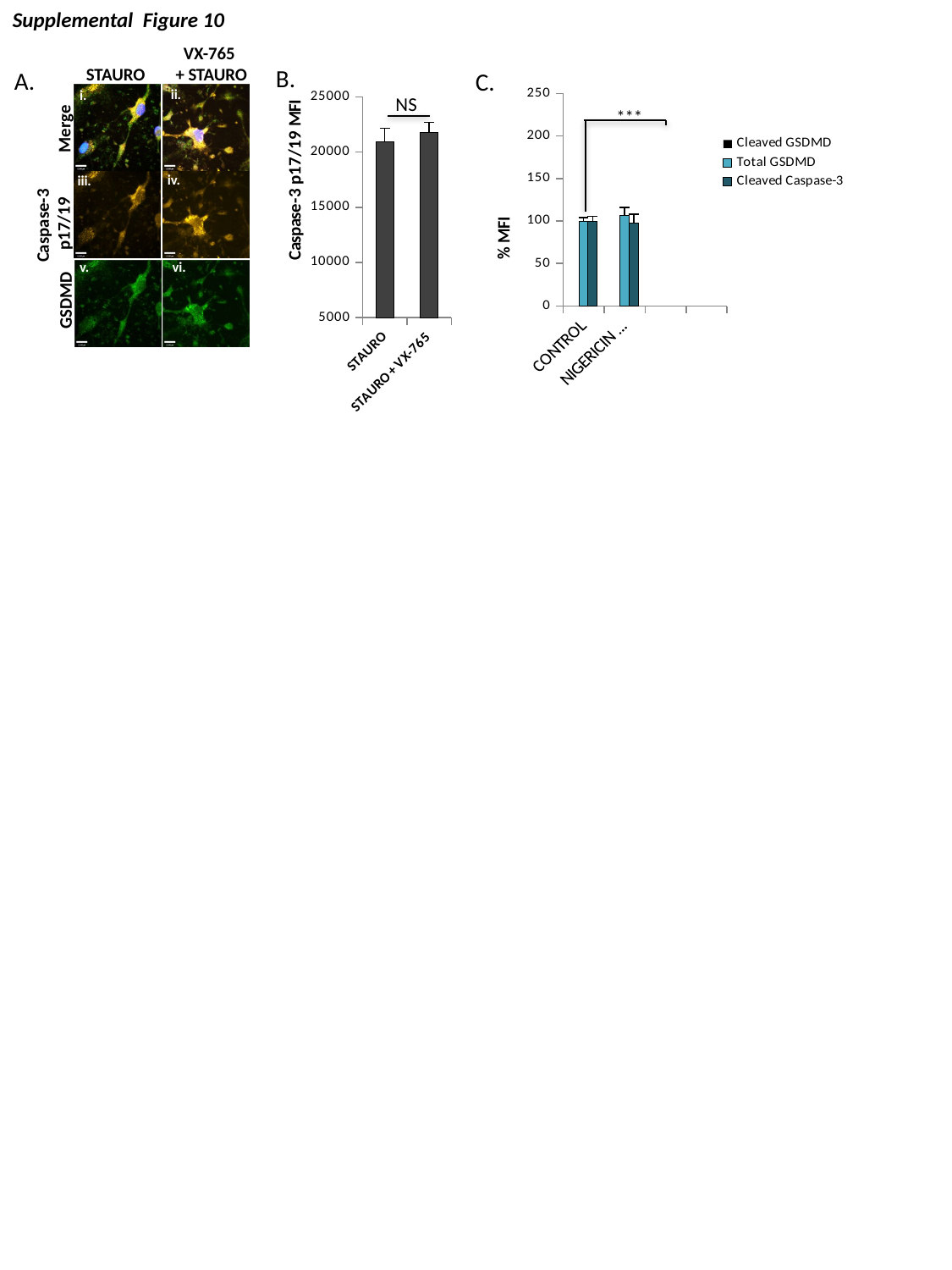

Supplemental Figure 10
VX-765
+ STAURO
STAURO
B.
A.
C.
### Chart
| Category | MEAN |
|---|---|
| STAURO | 20911.9 |
| STAURO + VX-765 | 21803.41304347826 |NS
### Chart
| Category | Cleaved GSDMD | Total GSDMD | Cleaved Caspase-3 |
|---|---|---|---|
| CONTROL | 100.0 | 100.0 | 100.0 |
| NIGERICIN 15 mins | 186.5633411478684 | 106.4500410669678 | 97.7896536195232 |ii.
i.
***
Merge
iv.
iii.
Caspase-3
p17/19
v.
vi.
GSDMD
